# Valorisation of plastic waste via metal-catalysed depolymerisation

**DOI:** 10.3762/bjoc.17.53

**Published:** 2021-03-02

**Authors:** Francesca Liguori, Carmen Moreno-Marrodán, Pierluigi Barbaro

**Affiliations:** 1 Consiglio Nazionale delle Ricerche, Istituto di Chimica dei Composti Organo Metallici, Via Madonna del Piano 10, 50019 Sesto Fiorentino, Firenze, Italy

**Keywords:** catalysis, depolymerisation, plastic, recycling, sustainable

## Abstract

Metal-catalysed depolymerisation of plastics to reusable building blocks, including monomers, oligomers or added-value chemicals, is an attractive tool for the recycling and valorisation of these materials. The present manuscript shortly reviews the most significant contributions that appeared in the field within the period January 2010–January 2020 describing selective depolymerisation methods of plastics. Achievements are broken down according to the plastic material, namely polyolefins, polyesters, polycarbonates and polyamides. The focus is on recent advancements targeting sustainable and environmentally friendly processes. Biocatalytic or unselective processes, acid–base treatments as well as the production of fuels are not discussed, nor are the methods for the further upgrade of the depolymerisation products.

## Review

### 1. Introduction

In a circular-economy perspective, wastes are deemed precious feedstock usable in the production of fertilisers, fuels, chemicals and a variety of materials for packaging, housing, transport and clothing [1–2]. A considerable fraction of the waste currently produced by our society is due to plastics, which is a major problem [3–4]. Plastics are usually synthetic polymers recalcitrant to decomposition, and hence liable to accumulate in landfills or the environment when discarded [5–6]. Not all plastics can be reused, and thus having limited economic value [7–8]. Plastics may release toxic compounds dangerous to human health and the habitat [9–10]. Plastic materials are ubiquitous in our everyday life, which accounts for a global production of plastics of around 360 million tons in 2018 [11], of which more than 60% are disposed [12–13]. As a consequence, pollution from plastics is impressive, resulting in the diffusion of microplastics into soil [14–15], oceans [16–17], crustaceans [18] and rain [19].

Valorisation of plastic waste, via chemical conversion into reusable building blocks, may contribute to solving these problems while representing a strategy to reduce the carbon footprint of the chemical industry [20–21]. The approach deepens the concept of plastic recycling [22–23], first of all requiring a careful design of efficient and controlled depolymerisation processes. Despite of this hurdle, the implementation of effective plastics value chains through recovery, reprocessing and upgrade would be a tangible mean to turn a challenge into an opportunity [24–25]. The waste-to-products strategy is already in place for both animal [26–27] and plant biomass polymeric waste [28–29], for which mature technologies are operative [30].

When referring to polymers in general, there is often a lack of univocal definitions, which may lead to confusion between terminologies used as synonyms, though they are not [31–32]. The IUPAC recommendations provide a useful reference to this aim [33]. Thus, “degradation” is a broad term describing the “progressive loss of the performance or of the characteristics of a substance" due to the action of chemical (acids, air, halogens, solvents) or physical agents (heat, light). For polymers, the properties involved are, for instance, tensile strength, colour or shape, the change of which is usually associated with a modification of the chemical composition (e.g., as a consequence of oxidation, cross-linkage, bond cleavage). The term “biodegradation” indicates a “degradation caused by enzymatic processes resulting from the action of cells”. Although commonly used, also for artificial polymers, the term “biodegradable” specifically refers to biorelated polymers (i.e., proteins, nucleic acids, polysaccharides), which are "susceptible to degradation by biological activity by lowering of the molar masses of macromolecules". Therefore, in this situation, “chain cleavage” and “degradation” are used interchangeably. To avoid confusion, instead of “(bio)degradation”, in the present review, we will use the term “depolymerisation” to identify the “process of converting a macromolecule into (recoverable) monomers or a mixture of monomers". Another relevant definition is that of “bioplastic”, meaning “biobased polymer derived from the biomass or issued from monomers derived from the biomass”, wherein “biobased” indicates “composed or derived in whole or in part of biological products issued from the biomass”. Also, there is no universally accepted definition for “compostable”, as it differs between diverse issuers [34]. The criteria indicated by the European Commission through the standard EN 13432 “Packaging–Requirements for packaging recoverable through composting and biodegradation” include disintegration (i.e., breakdown of material to particles of a defined size), biodegradability, absence of negative effects on the composting process and amount of heavy metals below given maximum values [35–36].

**Figure 1:**
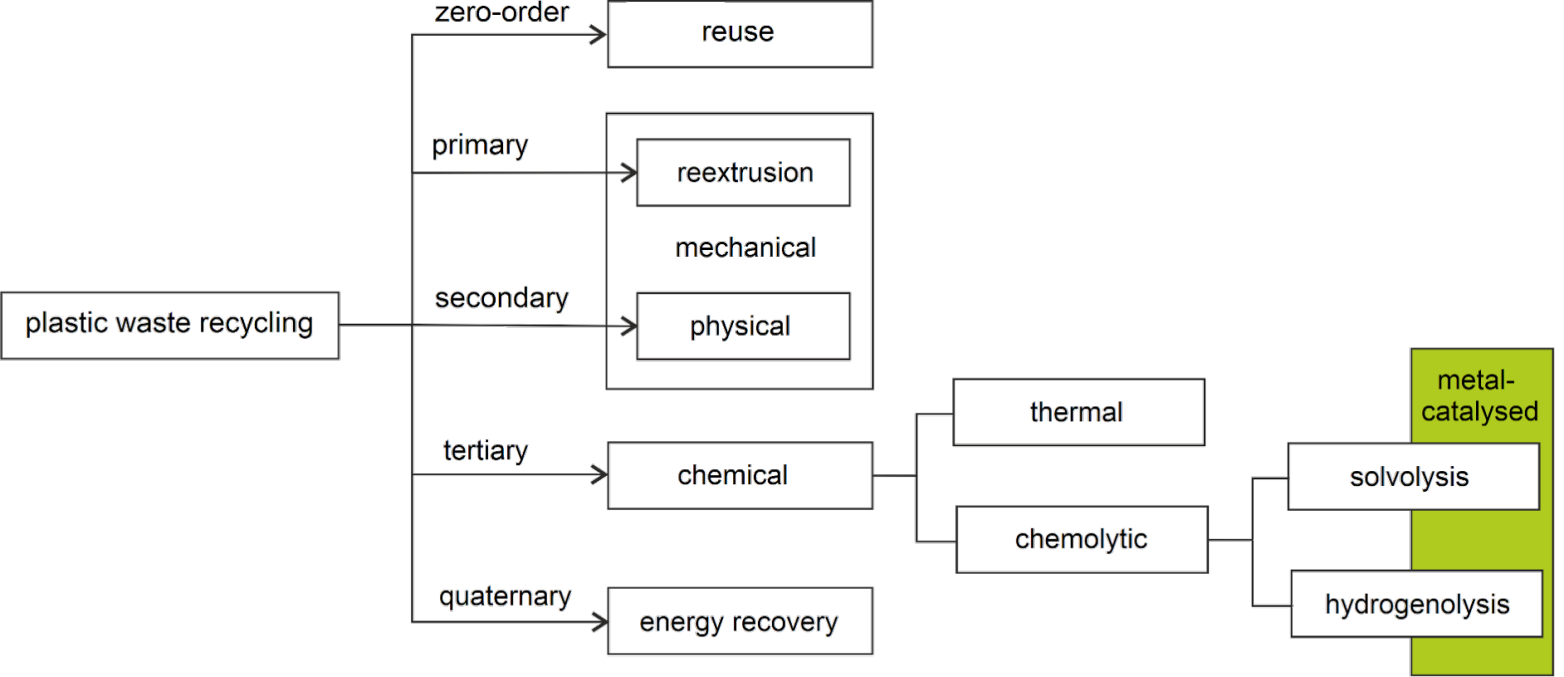
Potential classification of plastic recycling processes. The area covered by the present review is highlighted in green.

“Recycling” itself is a general term for which multiple definitions exist, depending on the year and the author [37]. Plastics manufacturers have also delivered their own guidelines under the name “Design for Recycling” [38–39]. A generally accepted definition for plastic recycling is “the process of recovering scrap or waste plastics and reprocessing the material into useful products, sometimes completely different in form from their original state” [40–41]. A possible classification of reported plastic recycling techniques is schematically shown in Figure 1 [42], wherein breakdown by the recycled polymer, the final product or the process involved further differentiates between methods, which may lead to occasional overlaps and inconsistencies [43–44].

“Reuse” is considered a zero-order recycling option, meaning “any operation by which products or components that are not waste are used again for the same purpose for which they were conceived” [45]. A typical example are plastic containers that are washed and reused as they are [46]. True recycling includes “any recovery operation by which waste materials are reprocessed into products, materials or substances whether for the original or other purposes. It includes the reprocessing of organic material but does not include energy recovery and the reprocessing into materials that are to be used as fuels” [45]. Therein, three options can be distinguished. “Reextrusion” (also known as closed-loop recycling) refers to the recycling of clean, uncontaminated single-polymer materials to give products with analogous performance and applications [47]. For example, polyethylene bottles are recycled into new bottles. Similarly, in “physical recycling”, the polymeric structure of the original material is maintained, although purification steps, the addition of additives or blending with fresh polymers may be included [48]. For this reason, both reextrusion and physical recycling are also referred to as “mechanical recycling”, primary and secondary, respectively [49–50]. However, materials of lower quality and economic value can be obtained [51–52]. By contrast, the term “chemical recycling” (or feedstock recycling) refers to those processes involving an alteration of the polymeric chain due to breakage of chemical bonds [53–54]. This definition may be confusing since chain scission may actually occur using either physical (heat) or chemical agents. Indeed, chemical recycling processes can be divided into two main categories: thermochemical and chemolytic routes. All of these processes may result in a variety of valuable products and the mixture thereof, including C1 molecules (CO, CO_2_, CH_4_), H_2_O and H_2_ due to complete decomposition of the polymeric chain, monomers or oligomers, depending on the waste polymer and the process. The as-obtained compounds can be reused as raw materials for the process industry (hence the term feedstock) to produce chemicals, fuels or other polymers. Thermochemical processes include pyrolysis [55], catalytic cracking [56] and gasification [57]. These are usually unselective, high-temperature treatments (300–1000 °C) that may efficiently provide light hydrocarbons or small molecules [58–59]. Chemolytic processes, wherein a chemical reagent is used to achieve depolymerisation, mainly involve solvolysis (a solvent is the reagent and solvolyses include hydrolysis, glycolysis, alcoholysis and aminolysis) and hydrogenolysis reactions (H_2_ as reagent). Hydrolysis (sometimes called hydrocracking) is in between thermochemical and chemolytic processing, basically consisting of depolymerisation by the combined action of heat and dihydrogen [60]. Chemolytic processes may or may not be catalytic. They will be discussed in detail in the following section. A fourth option is “energy recovery”. Strictly speaking, this cannot be considered as recycling, consisting in the recovery of the energy contained in a material rather than the material itself, and it is usually achieved by combustion or incineration [61–62]. This method is generally used for plastics that cannot be economically recycled by other means [63–64]. However, it often entails the emission of toxic volatile compounds (furans, dioxins) [65]. The tar obtained may be used for road construction [66].

The present paper shortly reviews the most significant contributions that appeared in the literature, from January 2010 to January 2020, in the field of metal-catalysed selective depolymerisation of plastics to reusable monomers, oligomers or added-value chemicals (see Figure 1). Scientific achievements will be described according to the plastic substrate, irrespective of the metal catalyst. Uncatalysed depolymerisations, full chain-cracking or unselective processes, acid–base treatments, as well as the production of fuels from plastics, will not be covered. Conversion of plastic waste to fuels [67–68] and biocatalytic depolymerisation methods [69–70] have been extensively and recently reviewed elsewhere, hence they will not be considered herein.

### 2. Depolymerisation of plastics

As outlined above, depolymerisation of plastic waste to reusable building blocks is an attractive option for effective recycling and valorisation. This is most conveniently achieved through chemolytic processes because of the higher selectivity and lower energy inputs compared to thermochemical approaches [71]. This is a “hot” research topic, mainly due to the few industrial applications developed in the field so far, despite the urgent need for innovative technologies that overcome the high costs of recycling, the legal constraints for dumping, the accumulation of plastic scraps and the dependence on nonrenewable (fossil) sources [72–73]. A reason for this underdevelopment is that chemolysis of plastics is still challenging due to multiple critical factors: i) the achievement of selective depolymerisation is only possible by carefully controlled reaction conditions, ii) the related processes must be “green” and economically viable and, iii) tailored solutions are required to overcome the chemical inertness for, and the thermodynamic limitations of the reversal of each polymer. Indeed, the ease (and outcome) of chain scission does not depend on the origin of the polymer but on its chemical structure [74–75]. For instance, plastics derived from biomass are not necessarily biodegradable, particularly if similar to those obtained from petroleum sources [76–77]. For depolymerisation to be effective at reasonable operating temperature and selective, the plastic substrate should in principle originate from low-exergonic polymerisation reactions [78]. This justifies for the easier depolymerisation of polyesters and polycarbonates compared to polyolefins [79–80]. By contrast, poor selectivity and slow kinetics of depolymerisation can be circumvented using a catalyst.

#### 2.1 Chemolysis

Several catalytic depolymerisation processes of plastics have been developed, using a solvent or molecular hydrogen as cleaving agents [81]. The main solvolytic process include:

- hydrolysis (water)
- alcoholysis (methanol, ethanol, 1-butanol, 2-ethyl-1-hexanol, phenol)
- glycolysis (ethylene glycol (EG), 1,2-propanediol (PD), 1,3- and 1,4-butanediol (BD)), diethylene glycol (DEG), dipropylene glycol (DPG))
- aminolysis (2-aminoethanol, 3-amino-1-propanol, ethylenediamine).

**Figure 2:**
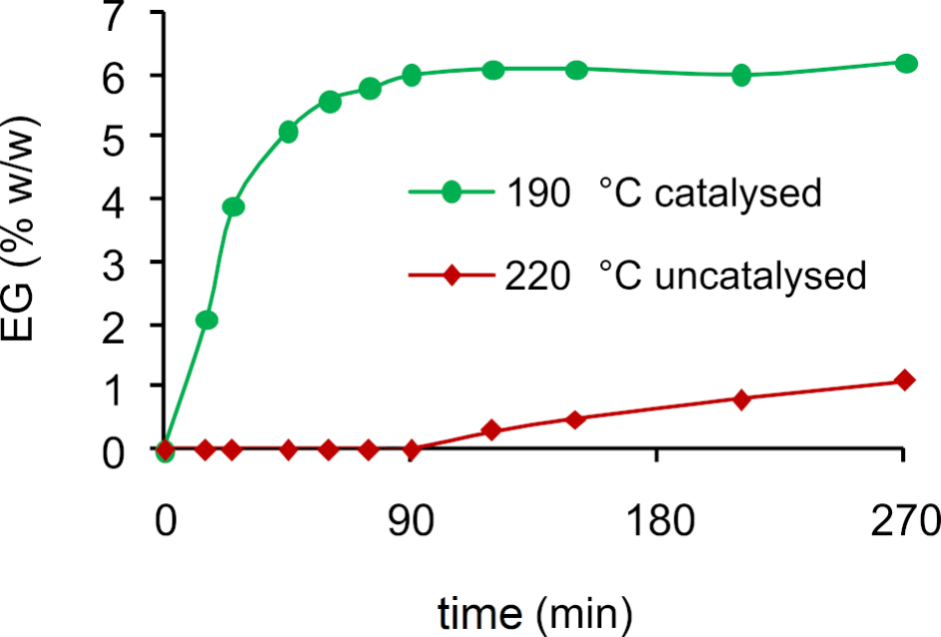
EG produced during glycolytic depolymerisation of PET using DEG + DPG as solvent and titanium(IV) *n*-butoxide as catalyst. Data from reference [92].

Advantages of catalytic processes are obvious and can be witnessed in the hydrolysis and the glycolysis reactions of poly(ethylene terephthalate) (PET) [82–83]. Representative data are reported in graphical format in Figure 2 for the glycolysis reaction of PET, using titanium(IV) *n*-butoxide as the catalyst. Compared to the uncatalysed process, benefits include milder reaction conditions, higher selectivity and productivity and reduced generation of waste; in short, improved sustainability [84–85]. However, solvolytic methods are usually not cost-competitive and energy-intensive [86], while they may involve the management of large amounts of noxious solvents and a variety of (decomposition) byproducts [87–88]. Depolymerisation products of course depend on the polymer, the solvent and the reaction conditions. For instance, for polyesters, alcoholysis may provide mixed monomers formally derived from transesterification reactions [89–90], while aminolysis provides amides and alcohols [91–92].

In the search of “greener” technologies for plastic recycling, catalytic hydrogenolysis processes have been developed that benefit from the use of H_2_ as clean reagent and that usually result in a limited number of secondary products [93–94]. The excess of H_2_ reagent can also be easily removed from the reaction mixture. The approach, referred to as hydrodeoxygenation [95–96], is already in use for the valorisation of naturally-occurring polymeric waste, i.e., lignocellulosic biomass [97–98], particularly lignin [99–100] and cellulose [101], to monomers or added-value platform molecules [102–103]. Here the main drawbacks concern safety hazards, supply, transport and storage costs of hydrogen. Catalytic transfer hydrogenation (CTH) methods from safer reagents have thus been developed and successfully applied to lignocellulose polymers [104–105].

#### 2.2 Catalysts

Catalysts of various types, including homogeneous and heterogeneous, have been reported for the above-mentioned depolymerisation processes of plastics. Heterogeneous systems are preferred by industry due to the easier separation from the reaction mixture, reuse and integration into existing reactor equipment [106–107]. Metal-based catalysts have been used for both solvolytic and hydrogenolytic methods, wherein the latter are usually achieved by supported metal species (Ru, Ir), due to the ability to activate molecular hydrogen, functioning as redox centres. The mechanisms of the metal-catalysed solvolytic reactions of plastics are all very similar and typical of conventional organic processes: a metal ion acts as Lewis acid centre for the activation of the chain-linking group of the polymer (either an ester, carbonate, ketone or amide) toward the nucleophilic attack of the various solvents. Specific examples, broken down according to the nature of the polymer and the process, will be reported in the next sections, in which metal catalysts are described in detail. Solvolytic depolymerisations can also be promoted by metal-free soluble acid or base catalysts. However, concentrated solutions, quasistoichiometric amounts or strong mineral acids (HNO_3_, H_2_SO_4_, H_3_PO_4_) or bases (NaOH, KOH, potassium butoxide) are often required, particularly for hydrolysis reactions [108–109], which may result in corrosion problems, troublesome neutralisation and purification procedures as well as a considerable generation of waste, which makes these process economically and environmentally unappealing [110–111]. These systems are not considered in the present review.

Biological catalysts for the deconstruction of plastics were extensively studied in the past years, and several hydrolytic-enzymes-containing microorganisms have been shown to be usable for this purpose [112–113]. However, enzymatic depolymerisation is hampered by high molecular weight and crystallinity, reduced chain mobility and hydrophobicity of polymers [114–115], which makes biodegradation often ineffective and time-consuming, particularly for polyolefins, such as polyethylene, polyvinyl chloride (PVC), polystyrene or PET [116–117]. Thus, abiotic pretreatments may be required, including UV irradiation [118], oxidation [119] or acidic degradation [120].

It is worth mentioning that organocatalytic depolymerisation methods have also been reported [121–122]. Despite these systems represent promising “greener” options, they still are in an early development stage. Uses are mainly limited to nitrogen-based catalysts, ionic liquids [123–124] and alcoholysis of oxygen-containing polymers, such as polyesters, polycarbonates and polyamides, i.e., glycolysis of PET, wherein high temperatures and nearly stoichiometric amounts of catalysts are often required to achieve moderate yields of monomers [125–126].

### 3. Selective depolymerisation of plastics via metal catalysis

Research in depolymerisation of plastics by artificial metal catalysts is relatively recent as most of the earliest studies are related to biocatalytic systems. Metal salts are the conventional catalysts for these processes, wherein acetates, phosphates and chlorides of heavy metals (Ti, Zn, Mg, Co, Fe) or lead oxide are commonly used in the alcoholysis and glycolysis of polyesters [127–128]. Despite that these systems ensure high conversions and selectivity, shortcomings relate to the harsh reaction conditions, slow kinetics, cost of metals, toxicity, difficulty in catalyst reusing and need of downstream processing. Significant efforts have thus been made to develop greener and sustainable catalytic systems featuring high efficiency under mild conditions. The use of sodium carbonate or bicarbonate as ecofriendly catalyst replacements for zinc acetate in the glycolysis of PET are examples of this direction [129–130]. Recent studies focused on molecular complexes as homogeneous catalysts, whereas heterogeneous systems based on solid-supported metal nanoparticles (NPs) have been scarcely investigated.

#### 3.1 Polyolefins

Due to the intrinsic chemical resistance of the hydrocarbon skeleton devoid of functional groups, polyolefins are neither prone to chemical recycling nor biodegradable [131–132]. Hence, they are more commonly repurposed via mechanical recycling, burned or just discarded [133–134]. Depolymerisation of polyolefins usually requires thermal treatments at high temperature [54,135].

**3.1.1 Polyethylene (PE):** PE is the most used thermoplastic material today, having a variety of uses in several fields. Applications of PE depend on the mechanical and physical properties (particularly the tensile strength, hardness and melting point *T*_m_), which are, in turn, ruled by the molecular weight and degree of branching [136–137]. Various types of PE exist, which are classified according to the density, the most common being high-density polyethylene (HDPE) and low-density polyethylene (LDPE). HDPE (0.94–0.97 g⋅cm^−3^, *T*_m_ 130 °C) is a highly crystalline material with a low degree of short chain branching. Owing to the high stiffness, tensile strength, resistance to moisture and gas permeability, it is mainly used in the manufacture of water pipes, toys, beverage bottles, outdoor furniture, housewares and electrical cables [138–139]. LDPE (0.91–0.94 g⋅cm^−3^, *T*_m_ 120 °C) is a poorly crystalline material having a high degree of short chain and long chain branching. It is featured by a good flexibility, transparency and high impact strength, which make it suitable for short-term applications, such as films, food packaging, squeezable bottles, plastic bags and medical devices [140]. PEs (except cross-linked samples) are partially soluble in (hot) aromatic hydrocarbons or in chlorinated solvents [141].

Depolymerisation of PE by catalytic pyrolysis or cracking into liquid fuels was recently reviewed [67,142]. Most of these processes are promoted by heterogeneous acid catalysts (e.g., zeolites, alumina, silica) and are usually unselective, resulting in a broad distribution of gas (C_3_ and C_4_ hydrocarbons), liquids (cycloparaffins, oligomers, aromatics) and solid products (char, coke) as a consequence of the random scission of C–C bonds into radicals, which leads to a complex mixture of olefinic and cross-linked compounds [143].

In a few cases, good selectivity to a liquid fraction was achieved. For instance, nanostructured BaTiO_3_ doped with Pb provided a mixture of liquid products, which includes alkanes (73.4%), olefins (22.5%) and naphthalene (4.1%) at total HDPE conversion at 350 °C [144]. In another example, hydrocracking of PE was performed over Pt NPs supported on SrTiO_3_ perovskite nanocuboids [145]. Virgin PE (*M*_w_ = 18000–420000 g⋅mol^−1^) or PE from single-use plastic bags (*M*_w_ = 115000 g⋅mol^−1^) was converted in >97% yield into liquid hydrocarbon (alkane) products having a narrow distribution of the molecular weight (960–1130 g⋅mol^−1^) under 11.7 bar H_2_ at 300 °C and solvent-free conditions [146]. The pyrolysis oils produced may be used as lubricants, waxes or further processed into detergents and cosmetics [147]. The catalyst could be recycled, however, with reduced performance due to Pt nanoparticle oxidation.

**Scheme 1:**
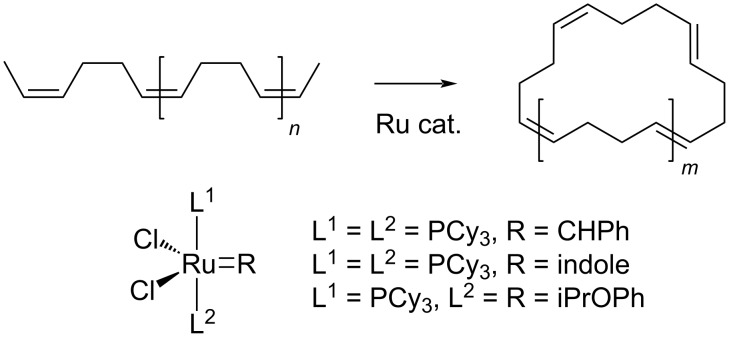
Simplified representation of the conversion of 1,4-PBD to C_16_–C_44_ macrocycles using Ru metathesis catalysts.

**3.1.2 Polybutadiene (PBD):** Partial depolymerisation of 1,4-PBD (*cis*, *trans*, *M*_w_ 1800–500000 g⋅mol^−1^) was achieved by an unconventional tandem ring-opening–ring-closing metathesis route mediated by commercially available Ru homogeneous catalysts [148]. The process afforded C_16_ to C_44_ mixtures of macrocyclic oligobutadienes with up to 98% selectivity at moderate conversions (59–88%) using first-generation Ru complexes bearing a tricyclohexylphosphine (PCy_3_) ligand, mild reaction temperature (35 °C) but toxic CH_2_Cl_2_ solvent (Scheme 1). The reaction using second-generation N-heterocyclic carbene ligands was faster and preferably yielded cyclododecatriene. Larger cyclic butadienes may be used in the production of flame retardants, lubricants and specialty polymers [149–150].

**3.1.3 Polystyrene (PS):** PS is a low-cost, hard and brittle plastic used both as a solid or foam in protective packaging, containers and trays [151]. It is a nonbiodegradable material accounting for about 10% of municipal solid waste [135]. It is soluble in benzene, carbon disulfide, chlorinated hydrocarbons, lower ethers and *N*-methyl-2-pyrrolidone (NMP) and has a melting point around 240 °C [152]. Most methods for PS recycling are not economically advantageous [153]. Mechanical recycling, based on pelletizing and moulding, produces low-grade plastics with poor mechanical strength and low market value. Solid PS products, such as coffee cups or take-away containers, can be recycled into videocassette cases or office equipment. Incineration at a temperature below 1000 °C and insufficient air is believed to produce a mixture of volatile compounds, including hazardous polycyclic aromatic hydrocarbons, alkylbenzenes and benzoperylene [154–155]. As for other polymers, pyrolytic methods end up to be poorly selective.

In some cases, metal-catalysed depolymerisation processes of PS were described, showing significant selectivity. In an earlier example, thermal treatment of PS waste over a Fe–K@Al_2_O_3_ catalyst at 400 °C provided a hydrocarbon oil in 92% yield, 71.4% of which were attributable to styrene monomer [156]. A decrease of 56 kJ mol^−1^ for the activation energy of PS depolymerisation was calculated in the presence of the catalysts. More recently, high-porosity montmorillonite (Mt) was used to prepare Mg-, Zn-, Al-, Cu- or Fe-decorated heterogeneous catalysts [157–158]. An oil yield around 89% was obtained at 450 °C over 20% Fe@Mt, composed by 51%, 10% and 6% (wt) styrene, toluene and ethylbenzene, respectively, and additional oligomers.

#### 3.2 Polyesters

**3.2.1 PET:** PET is one of the most widely used thermoplastic polyesters, particularly in the textile and food packaging industry (e.g., soft-drink and water bottles, food container, films) due to the excellent thermal and mechanical properties, durability, inertness and transparency. The global production of PET exceeds 50 million tons pear year, while PET accounts for around 8% by weight and 12% by volume of the world's solid waste [159–160]. PET is a copolymer of terephthalic acid (TPA) and EG [161]. It is best soluble in chlorophenol, tetrachloroethane, *m*-cresol, NMP, nitrobenzene and 1,1,1,3,3,3-hexafluoro-2-propanol, insoluble in common alcohols and water and has a melting point of 250 °C and a glass transition temperature *T*_g_ of 76 °C [162–163]. It was suggested that under certain circumstances, PET may leach phthalates [164], which are known for potentially adverse health effects and subject to ECHA regulation restrictions [165–166]. Coupled with the fact that TPA is produced from petrochemical sources, bioderived 2,5-furandicarboxylic acid has been proposed as TPA replacement in the production of plastic bottles, representing one of the rare examples of industrial manufacture of biobased polymers [167–168].

**Figure 3:**
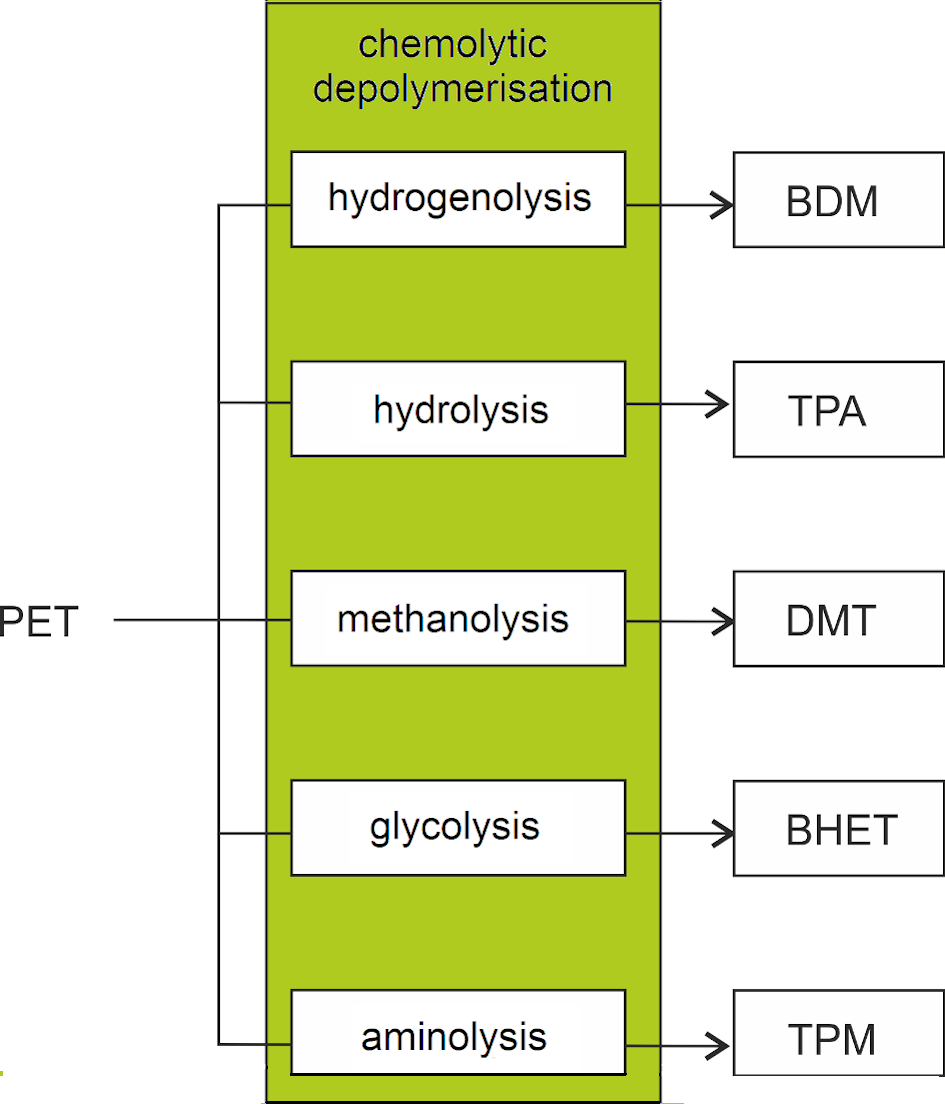
Main added-value monomers obtainable by catalytic depolymerisation of PET via chemolytic methods.

From the chemical recycling point of view, PET is one of the most studied plastics, so as to represent a case study in the field [169–170]. A variety of added-value, reusable chemicals and monomers can be recovered from PET via chemolytic depolymerisation, including 1,4-benzenedimethanol (BDM), TPA, dimethylterephthalate (DMT), bis(2-hydroxyethyl)terephthalate (BHET), terephthalamides (TPM [171]) and EG (Figure 3). Catalytic hydrogenolysis, hydrolysis, methanolysis and glycolysis reactions of (postconsumer) PET have been reviewed, each showing their own advantages and disadvantages [172–173]. For instance, glycolysis usually requires more problematic purifications than methanolysis, which, on the other hand, is generally more energy-intensive. Some solvolytic processes of PET are already in operation at the industrial or pilot scale [174–175]. However, they often rely on the use of considerable amounts of strong alkali bases and chlorinated solvents [176–177], which makes them neither economically competitive nor environmentally friendly [178–179]. A survey of patents related to the chemical recycling of PET up to 2005 can be found in the literature [180].

**Scheme 2:**
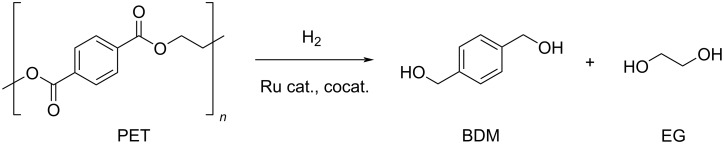
Hydrogenolytic depolymerisation of PET by ruthenium complexes.

Hydrogenolysis. In the recent years, hydrogenolysis reactions of PET were developed mostly using Ru metal-based catalysts, due to their higher affinity for C=O bond (ester) hydrogenation compared to other metals (Scheme 2) [181–182]. Thus, a 73% BDM yield was obtained using a soluble Ru(II)–PNN complex at 110 °C in THF/anisole solvent, 50 bar of hydrogen and a 20:1 excess of strong base potassium *tert*-butoxide as cocatalyst (Table 1, entry 1) [183]. Although the reaction mechanism was not investigated in detail, it was suggested that cleavage of the ester linkage may occur in a concerted manner through the reported heterolytic route [184–185]. The role of butoxide was postulated to be the activation of the heterogeneous splitting of dihydrogen. BDM is an important building block for the production of resins and polyesters other than PET [186–187]. Analogously, a similar Milstein-type ruthenium–PNN complex, generated in situ by treatment of the chloride catalyst precursor with potassium butoxide in a 2:1 molar ratio, resulted in a nearly quantitative yield of BDM and EG at a slightly higher reaction temperature (160 °C, 54 bar H_2_, Table 1, entry 2). Interestingly, PET flakes from postconsumer bottles could be used, showing the catalytic system to be resistant to the presence of contaminants and impurities (e.g., additives, pigments) [188]. More recently, effective PET depolymerisation was accomplished by a ruthenium molecular catalysts bearing the well-known tripodal phosphorous ligand 1,1,1-tri(diphenylphosphinomethyl)ethane (triphos) [189–190]. Thus, use of Ru(triphos)tmm (tmm = trimethylenemethane) and acidic bis(trifluoromethane)sulfonimide (HNTf_2_) cocatalyst (1:1) in noxious 1,4-dioxane solvent resulted in 41% PET conversion and 64% BDM selectivity at 140 °C and 100 bar H_2_ due to the formation of ether byproducts (Table 1, entry 3) [191]. No hypotheses for the reaction mechanism were formulated. Higher conversion (64%) and selectivity (99%) were observed using the bulkier xylyl derivative Ru(triphos-xyl)tmm (Table 1, entry 4), which was attributed to reduced catalyst degradation [192]. The catalyst could be employed for the depolymerisation of PET flakes from various sources (water bottles, dyed soda bottles, pillow filling, yoghurt pots). However, the role of HNTf_2_ was unclear.

**Table 1:** Hydrogenolysis of PET by soluble Ru molecular catalysts.

entry	catalyst	cocatalyst^a^	reaction conditions^b^			conv.^c^ (%)	BDM		ref.
	
			*T* (°C)	H_2_ (bar)	solvent		sel.^d^ (%)	TOF^e^ (mol_BDM_⋅mol_cat._^−1^⋅h^−1^)	

1	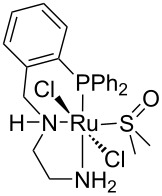	K*t-*BuO^f^	110	50	anisole/THF^g^	73	100	0.76	[183]
2	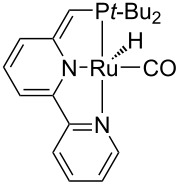	K*t-*BuO	160	54	anisole/THF^g^	99	100	1.03	[188]
3	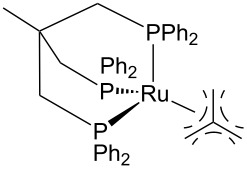	HNTf^h^	140	100	1,4-dioxane	42	64	1.68	[191]
4	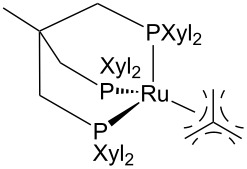	HNTf^h^	140	100	1,4-dioxane	64	99	3.96	[191]

^a^Catalyst loading 2 mol %, calculated based on repetition units in PET, cocatalyst molar ratio 2:1. ^b^Reaction temperature, hydrogen pressure, reaction time 48 h. ^c^PET conversion. ^d^Selectivity to BDM. ^e^Turnover frequency, calculated based on repetition units in PET and moles of Ru catalyst. ^f^Cocatalyst molar ratio 20:1. ^g^1:1 v/v. ^h^Catalyst loading 1 mol %. Reaction time 16 h.

**Scheme 3:**
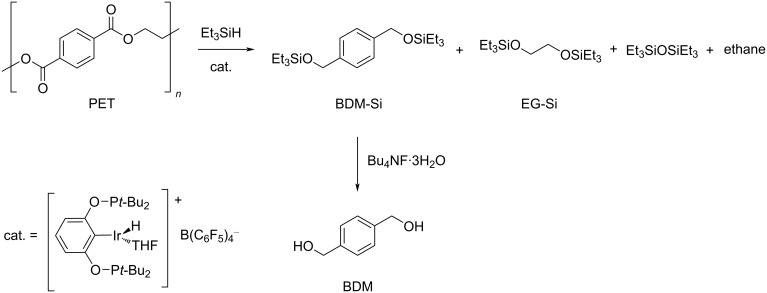
Depolymerisation of PET via catalytic hydrosilylation by Ir(III) pincer complex.

In a different approach, hydrogenolysis-like depolymerisation was achieved through a hydrosilylation strategy, using the pincer Ir(III) complex [Ir(POCOP)H(THF)][B(C_6_F_5_)_4_] (POCOP = 1,3-(*t*-Bu_2_PO)_2_C_6_H_3_) as catalyst and an excess of Et_3_SiH as reagent (chlorobenzene solvent, 70 °C) [193]. BDM could be obtained in an overall 58% yield from PET from fibres or bottles, after hydrolysis of the intermediate silyl ether using Bu_4_NF·3H_2_O in THF (Scheme 3).

**Scheme 4:**
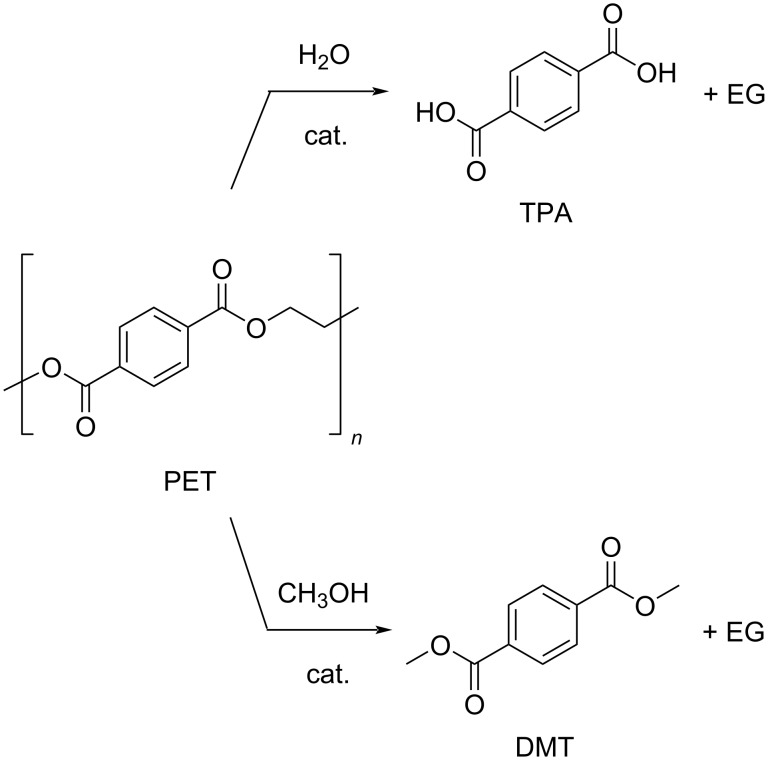
Catalytic hydrolysis (top) and methanolysis (bottom) reactions of PET.

Hydrolysis. Methods for the metal-catalysed hydrolysis of PET were developed, allowing for the recovery of costly TPA monomer (Scheme 4, top). TPA was obtained in 97.1% yield at full PET conversion, using 70 wt % aqueous ZnCl_2_ as catalyst at 180 °C and no organic solvent [194]. However, a high ZnCl_2_/PET weight ratio of 35 was required. The catalyst could be reused, showing significant activity decrease starting from the fourth cycle due to biochar formation. A mechanism was hypothesised in which Zn^2+^ ions act as a Lewis acid activator for the carbonyl ester bond. In a previous work, complete depolymerisation of PET was achieved using zinc acetate as catalyst in hot compressed water [195]. A TPA yield of 90.5% was obtained at 240 °C after 30 min reaction time, using a ZnAc_2_/PET weight ratio as low as 0.015. For this, a mechanism was speculated in which proton ions act as activators.

Methanolysis. To the best of our knowledge, only one example of a depolymerisation reaction of PET through alcoholysis was recently reported, which is however devoid of any catalysts [196]. Therein, a 97.3% yield of DMT was obtained at full PET conversion, by treatment of PET with methanol at 200 °C for 3.5 h (Scheme 4, bottom). No details of byproducts were provided. Ethanol and butanol were much less reactive under identical reaction conditions. The as-prepared DMT could be used for the production of hydrocarbon jet fuels by catalytic hydrogenation. Metal-catalysed methanolysis of PET was described in previous years [109,197].

**Scheme 5:**
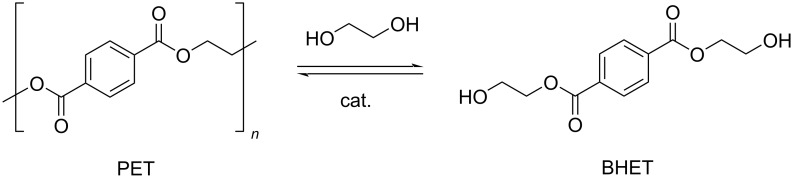
Depolymerisation of PET by glycolysis with ethylene glycol.

**Figure 4:**
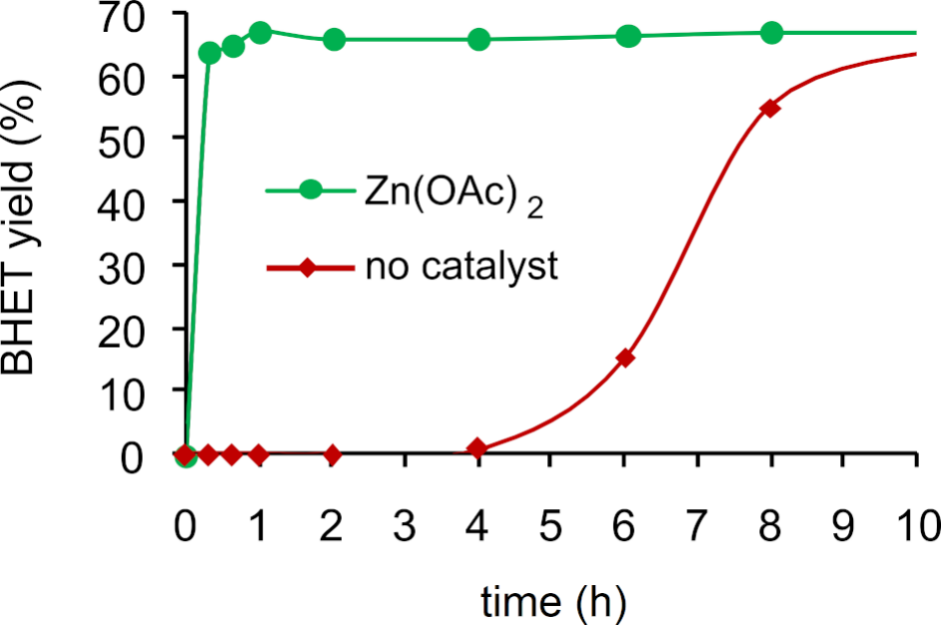
Glycolysis of PET: evolution of BHET yield over time, with and without zinc acetate catalyst (196 °C, EG/PET ratio 2.45 (w/w). Data from reference [129].

Glycolysis. Glycolysis is a convenient process for PET chemical recycling, owing to the low cost, relatively mild reaction conditions and the potential for the production of useful monomers and oligomers. These compounds can be used in the synthesis of recycled polyesters, polyurethanes, polyisocyanurates and resins [43]. Among the various glycols, EG is the most popular, resulting in the formation of BHET, formally through a (reversible) transesterification reaction (Scheme 5) [198]. Drawbacks of this method are the difficulty of purification of BHET, the need of an excess of EG and the possible product contamination by homogeneous catalysts [199]. The conventional catalysts for this reaction are EG-soluble metal acetates, the activity of which showed a decrease in the order Zn^2+^ > Mn^2+^ > Co^2+^, which was attributed to the diverse interaction between the metal cation promoter and the carbonyl group of polyester [200]. Indeed, several studies showed Zn-based catalysts to provide the best performances. A summary of recent findings and reaction conditions is reported in Table 2. In an earlier study, a 85.6% BHET yield was obtained at 196 °C, using an EG/PET ratio of 5:1 (w/w) and 1 wt % Zn(OAc)_2_ loading (Table 2, entry 1) [201]. The system resulted in partial selectivity to BHET due to the formation of significant amounts of oligomers, mainly BHET dimers, which increased upon standing. The kinetic of the zinc acetate-promoted process was studied over a range of reaction conditions, showing the best combination to be 196 °C, an EG/PET ratio of 2.45:1 (w/w) and a catalyst loading of 0.3 wt % (Table 2, entry 2) [129]. Under these conditions, an equilibrium yield of BHET around 65% was achieved within short reaction times (1 h), much faster than in the absence of catalysts or using alkali salt promoters (Na_2_CO_3_, NaHCO_3_, Na_2_SO_4_ or K_2_SO_4_, Figure 4). PET wastes, including highly coloured and multilayered PET, could be used as substrate. More recently, it was demonstrated that the addition of a cosolvent for PET, such as dimethyl sulfoxide (DMSO), NMP, nitrobenzene or aniline to the conventional PET-insoluble EG system, greatly enhanced the depolymerisation kinetics, resulting in improved conversions (the solubility of PET at *T* > 130 °C was aniline > NMP > nitrobenzene > DMSO) [202]. For instance, the use of a DMSO/EG 2:1, w/w solvent mixture resulted in an increase of PET conversion from 43.0% to 83.9% compared to pure EG (190 °C, 5 wt % catalyst loading, 5 min reaction time, Table 2, entry 4 vs entry 3). Using the same reaction conditions and solvent mixture, Mn, Co, Cu and Ni acetate catalysts were less active than Zn (Table 2, entries 5 and 6). In a further study, glycolysis of PET was performed under microwave heating in the presence of Zn(OAc)_2_, yielding BHET with an 80% selectivity at 97% conversion due to formation of dimers (Table 2, entry 7) [203–204]. Soluble metal chlorides (zinc, magnesium, iron, zirconium, cobalt, nickel) were also explored as catalysts in the glycolytic depolymerisation of PET [128,205]. The highest BHET yield (74.7%) was achieved using zinc chloride (0.5% w/w), an EG/PET ratio of 14:1 and reflux conditions (Table 2, entry 8). The use of preformed soluble Co(II) complexes bearing bidentate phosphorus ligands (e.g., 1,2-bis(dicyclophosphino)ethane) showed minimal improvements compared to the chloride salt catalyst [205]. The use of transition metal-substituted polyoxometallates (POMs), of the general formula K_6_SiW_11_MO_39_(H_2_O) (M = Zn^2+^, Mn^2+^, Co^2+^, Cu^2+^, Ni^2+^) [206–207], was also investigated [208]. The catalytic activity was found to decrease in the order Zn > Mn > Co > Cu > Ni, consistent with previous reports [209]. The best catalyst afforded BHET in 84.1% yield (Table 2, entry 9), which was rather constant over eight catalyst reuses [210]. A stepwise depolymerisation mechanism was proposed, via intermediate oligomers, in which Zn ions act as Lewis acid activators for the C=O ester bonds toward nucleophilic attack by EG.

**Table 2:** Catalytic EG glycolysis of PET by metal acetates or other soluble metal salts.

entry	material	catalyst	EG/PET^a^ (w/w)	*T*^b^ (°C)	conv.^c^ (%)	BHET		reference

						sel.^d^ (%)	TOF^e^ (mol_BHET_⋅mol_cat._^−1^⋅h^−1^)	

1	PET bottle chips	Zn(OAc)_2_	5:1	196	n.a.^f^	85.6^g^	27.0	[201]
2	PET waste	Zn(OAc)_2_	2.45:1	196	65	100	207.1	[129]
3	PET pellets	Zn(OAc)_2_	6:1	190	43.0	100	118.4	[202]
4	PET pellets	Zn(OAc)_2_	2:1^h^	190	83.9	100	230.9	[202]
5	PET pellets	Mn(OAc)_2_	2:1^h^	190	80.8	100	222.0	[202]
6	PET pellets	Co(OAc)_2_	2:1^h^	190	78.7	100	216.3	[202]
7	PET bottle flakes	Zn(OAc)_2_	5:1	196^i^	97.1	80.3	73.7	[203]
8	PET	ZnCl_2_	14:1	196	n.a.^f^	74.4^g^	n.a.^f^	[128]
9	PET pellets	Zn POM^j^	4:1	185	100	84.1	1292.3	[210]

^a^EG as solvent. ^b^Reaction temperature. ^c^PET conversion. ^d^Selectivity to BHET. ^e^Turnover frequency calculated based on PET repetition units and moles of metal catalyst. ^f^Not available. ^g^BHET yield (%). ^h^Solvent DMSO/EG 2:1, w/w. ^i^Microwave irradiation 500 W. ^j^Zn polyoxometalate formula K_6_SiW_11_ZnO_39_(H_2_O).

In addition to soluble catalysts, metal-containing insoluble materials, namely solid acid catalysts, were developed for the glycolytic depolymerisation of PET by EG. Representative data are summarised in Table 3, wherein the catalyst productivity is reported for comparison as mol_BHET_⋅g_cat._^−1^⋅h^−1^. Despite that both catalyst and PET were insoluble in EG at the reaction temperature, most systems displayed substantial activity. Thus, sulfated titania, zinc oxide and mixed oxides (SO_4_^2−^/TiO_2_, SO_4_^2−^/ZnO and SO_4_^2−^/ZnO–TiO_2_) were prepared, showing the amount of Lewis acid sites and the high surface area of the solid material to be critical in affecting the catalytic efficiency [211]. Best results were obtained for the binary oxide SO_4_^2−^/ZnO–TiO_2_ calcined at 200–300 °C (surface area 192 m^2^⋅g^−1^, density of acidic sites 4.34 mmol⋅g^−1^), which provided BHET in 73% selectivity at full PET conversion at 180 °C reaction temperature and a moderate excess of EG (5.5:1, w/w, Table 3, entry 1). The formation of a significant amount of oligomers was detected. The catalyst could be recovered by centrifugation and reused over four cycles with no efficiency decay. Similar effects were reported for Zn-substituted titanate nanotube (TiNT) catalysts [212–213]. Therein, a positive role of Zn^2+^ Lewis acid sites was demonstrated by the higher efficiency compared to the Na^+^ catalyst counterpart (Table 3, entry 2 vs entry 3), whilst a high surface area around 150 m^2^⋅g^−1^ was proposed to increase the number of exposed sites [214]. Zn@TiNT afforded BHET in 87% yield at 196 °C reaction temperature. TiNT have received significant general interest in heterogeneous catalysis because of the better active-site-accessibility compared to 2D materials, thanks to a typical 8–16 nm outer diameter tubular morphology [215–216] and the potential for facile metal doping via ion-exchange of the solid support [217–218]. Lewis acid-type catalytic activity was also postulated for γ-Fe_2_O_3_ NPs, which provided BHET in 90% yield at 300 °C (Table 3, entry 4) [219]. Therein, thanks to the superparamagnetic properties, easy recovery of the highly dispersed solid catalyst (11 nm size) was possible by application of a magnetic field. The catalyst could be reused over ten cycles without significant activity loss. Other solid-supported nanostructured metal oxides were tested as catalysts for PET glycolysis. Thus, a graphene oxide (GO)–Mn_3_O_4_ nanocomposite (Table 3, entry 5) [220] and silica NPs-supported Mn_3_O_4_ [221] resulted in a good yield of BHET (>90%), however, at a high reaction temperature. A zinc manganite spinel ZnMn_2_O_4_ gave BHET in 92% yield at 260 °C and 5 atm pressure (Table 3, entry 6) [222]. On the other hand, amphoteric solid catalysts have also shown usability in EG depolymerisation of PET. For instance, a BHET yield of 75% was achieved over (Mg–Zn)–Al-layered double hydroxides (LDH) catalysts at 196 °C (Table 2, entry 7) [223]. A cooperative mechanism was proposed in which Lewis acid sites (Mg^2+^, Al^3+^, Zn^2+^) activate the C=O ester bond, while the basic sites (OH^−^) deprotonate EG, enhancing the nucleophilic cleavage of the ester unit [224].

**Table 3:** Glycolysis of PET using EG and insoluble, solid-supported metal catalysts.

entry	material	catalyst	EG/PET (w/w)	*T*^a^ (°C)	conv.^b^ (%)	BHET		reference

						sel.^c^ (%)	productivity^d^ (mol_BHET_⋅g_cat._^−1^⋅h^−1^)	

1	PET pellets	SO_4_^2−^/ZnO–TiO_2_	5.5:1	180	100	73.0	0.42	[211]
2	PET bottle grains	Zn@TiNT	4:1	196	99	87.0	0.45	[214]
3	PET bottle grains	Na@TiNT	4:1	196	99	80.1	0.42	[214]
4	PET	γ-Fe_2_O_3_ NPs	3.7:1	300	100	90	0.09	[219]
5	PET granules	GO–Mn_3_O_4_	3.7:1	300	100	96.4	0.38	[220]
6	PET bottles	ZnMn_2_O_4_	5.5	260	100	92.2	0.48	[222]
7	PET pellets	(Mg–Zn)–Al LDH	10:1	196	100	75.0	0.13	[223]

^a^Reaction temperature. ^b^PET conversion. ^c^Selectivity to BHET. ^d^Catalyst productivity calculated based on PET repetition units, moles of BHET and grams of solid catalyst.

**Scheme 6:**
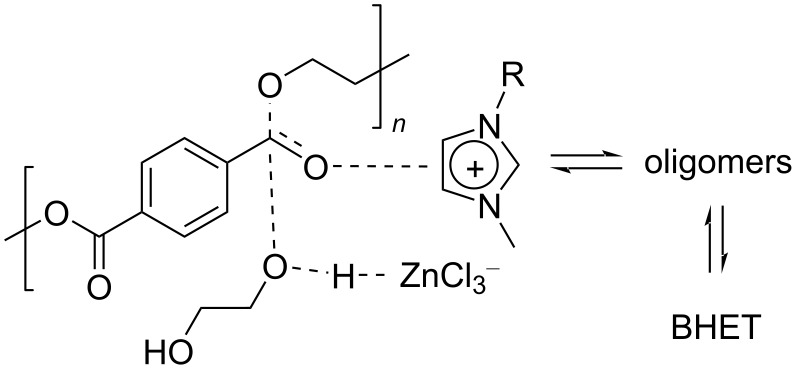
Potential activated complex for the glycolysis reaction of PET catalysed by metallated ILs and evolution toward products.

Notably, the depolymerisation of PET by EG was also reported using metal-containing catalysts in the form of ionic liquids (ILs) [209]. Advantages of metallated ILs include low flammability, high thermal stability and versatility. However, their “greenness” and toxicity are still debated [225–226]. Thus, amim[ZnCl_3_] (amin = 1-allyl-3-methylimidazolium, Table 4, entry 1) [227] and amim[ZnCl_3_] (bmim = 1-butyl-3-methylimidazolium, Table 4, entry 2) [228] were recently studied, showing higher catalytic activity compared to metal-free ionic liquids (i.e., bmim chloride), the conventional catalysts (i.e., ZnCl_2_, Zn(OAc)_2_) or the analogous Mn, Co and Cu ionic liquids. Typically, 80–85% BHET yields were observed for metallated ILs, while under the same experimental conditions, ZnCl_2_ gave BHET in ≈70% yield (Table 4, entry 5). Similar results were reported for the bmim_2_[MCl_4_] (M = Cr, Fe, Co, Zn, Ni, Cu) catalysts, wherein the cobalt derivative resulted in the best performance (Table 4, entry 3) [229]. Based on infrared studies, the higher catalytic activities of the metallated ILs was attributed to their higher Lewis acidity compared to both the metal-free catalysts and the metal salts. A mechanism was therefore proposed in which a synergistic effect of the metallated ILs takes place, based on the activation of the C=O bond by the IL Lewis acid cation and of the hydroxy group in EG by the IL anion (Scheme 6). The IL catalysts could be easily separated by distillation and reused up to six times with no significant efficiency drop. Catalytic glycolysis by heterogenised, metallated ionic liquids was also investigated [230], however, showing a lower performance compared to the soluble systems. Thus, PET pellets were fully converted by a bmim[Fe(OAc)_3_] catalyst immobilised onto bentonite, affording BHET in 44% yield (Table 4, entry 4) [231]. The solid catalyst could be recovered by filtration and reused.

**Table 4:** Catalytic EG glycolysis of PET using metallated ionic liquids.

entry	catalyst		EG/PET (w/w)	*T*^a^ (°C)	conv.^b^ (%)	BHET		reference
	
	formula	loading (wt %)				sel.^c^ (%)	TOF^d^ (mol_BHET_⋅mol_cat._^−1^⋅h^−1^)	

1	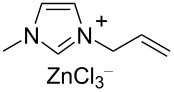	10	4:1	175	100	80.1	9.8	[227]
2	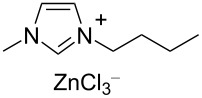	1.25	11:1	190	100	84.9	55.2	[228]
3	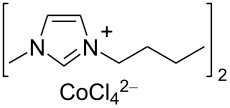	17	12:1	175	100	81.1	8.5	[229]
4	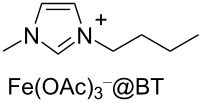	30^e^	7:1	190	100	44.0	2.9^f^	[231]
5	ZnCl_2_^g^	1.25	4:1	175	94	76.2	4.0	[227]

^a^Reaction temperature. ^b^PET conversion. ^c^Selectivity to BHET. ^d^Turnover frequency calculated based on PET repetition units and moles of metal catalyst. ^e^Loading of bmim[Fe(OAc)_3_] immobilised onto bentonite. ^f^Calculated with respect to the moles of iron. ^g^Metal salt.

As an alternative to ILs, metal-based deep eutectic solvent (DES) systems were also explored as catalysts for the glycolysis reaction of PET using EG. DES have similar properties to metallated ILs, but they are cheaper and less toxic [232–233]. Because of this, they have found application in many fields [234–235], though they cannot be considered inherently “green” [236]. In a recent work, the DES combination zinc acetate and 1,3-dimethylurea (1:4) showed the highest catalytic activity among a series of transition metal acetates (Zn, Mn, Co, Ni, Cu), affording BHET with 82% yield and noticeable productivity (TOF = 129 h^−1^, based on the moles of Zn) at 190°C, EG/PET 4:1, w/w and 5 wt % catalyst [237]. A mechanism was proposed analogous to that depicted in Scheme 6, but in which Zn^2+^ acts as Lewis acid and dimethylurea acts as base promoter for EG hydroxy deprotonation [238]. The remarkable activity was attributed to the dual effect of base and acid catalysis, in addition to the solubility of the catalyst in EG.

**Scheme 7:**
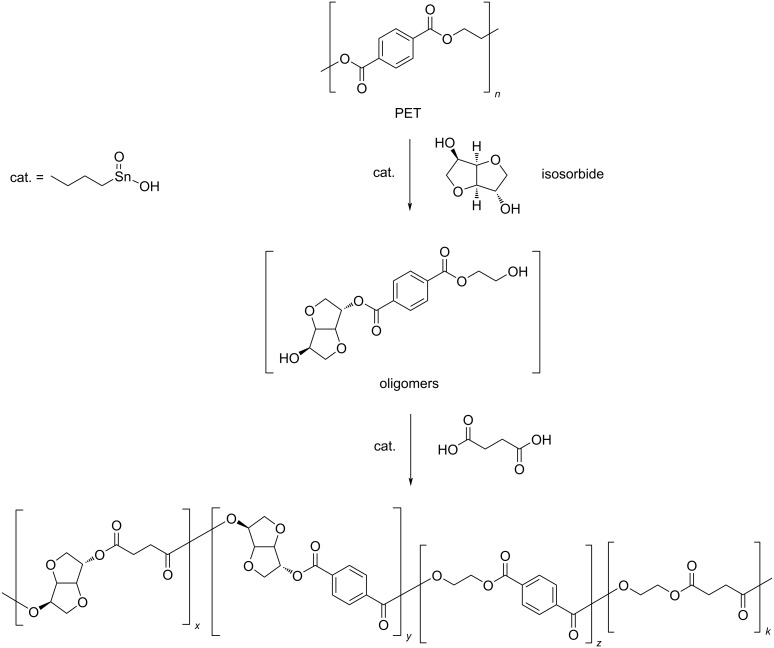
One-pot, two-step process for PET repurposing via chemical recycling.

It is worth mentioning that, in addition to the recovery of chemicals via chemolytic processes, repurposing techniques of PET were developed based on one-pot, two-step glycolysis-reprocessing strategies, wherein the depolymerisation products are directly used in a polymerisation reaction, without intermediate purifications. In order to avoid the presence of free glycols in the polymerisation mixture, most of these processes were performed using (sub)stoichiometric amounts of diol cleaving agents, other than EG (for instance, PD [239], DEG [240–241]). As a consequence, the depolymerisation step usually results in complex mixtures of oligomers. Moreover, reacting diols may be unstable under the reaction conditions adopted. Hence, if used in excess, a significant formation of decomposition byproducts may be observed (i.e., dioxane and acetaldehyde for DEG [242]). Because of that, these metal-catalysed depolymerisations cannot be strictly considered as selective, although the overall processes are interesting from a practical and sustainability point of view. Some recent examples are cited herein. Postconsumer PET was depolymerised in the melt (at 250 °C) using DEG and Ca/Zn stearate as catalyst, and the product mixture was used in situ in conjunction with bis(2-ethylhexyl)phthalate and the same metal promoter for the production of flexible poly(vinyl chloride) compounds [243]. One-pot depolymerisation–polycondensation reactions were developed to produce random copolyesters poly(ethylene terephthalate-*co*-adipate) from PET in the presence of EG and adipic acid [244]. The depolymerisation step was carried out using a zinc acetate catalyst (1 wt %), with no need of excess of chemicals. Polymerisation was then achieved by raising the reaction temperature, without purification of the intermediate oligomers being required. An interesting one-pot process was developed that combines the use of bioderived chemicals, isosorbide and succinic acid, with PET chemical recycling to produce novel polyesters [245]. In this process, isosorbide was used as depolymerising diol to give a mixture of differently composed oligomers, whereas succinic acid was added in the second step as polymerising comonomer (Scheme 7). Both steps were efficiently catalysed by monobutyltin oxide, using substoichiometric amounts of isosorbide and succinic acid and no solvent at 230 °C reaction temperature. Isosorbide is a safe chemical [246] that is obtainable on the large scale from renewable glucose [247–248]. Because of this and due to the inherent rigid structure, conferring the resulting polymers with excellent mechanical properties (e.g., stiffness, toughness, hardness), isosorbide is used as monomer in the production of a variety of plastics [249–250]. By contrast, the rigidity results in a poor reactivity as depolymerising agent [251].

**Scheme 8:**
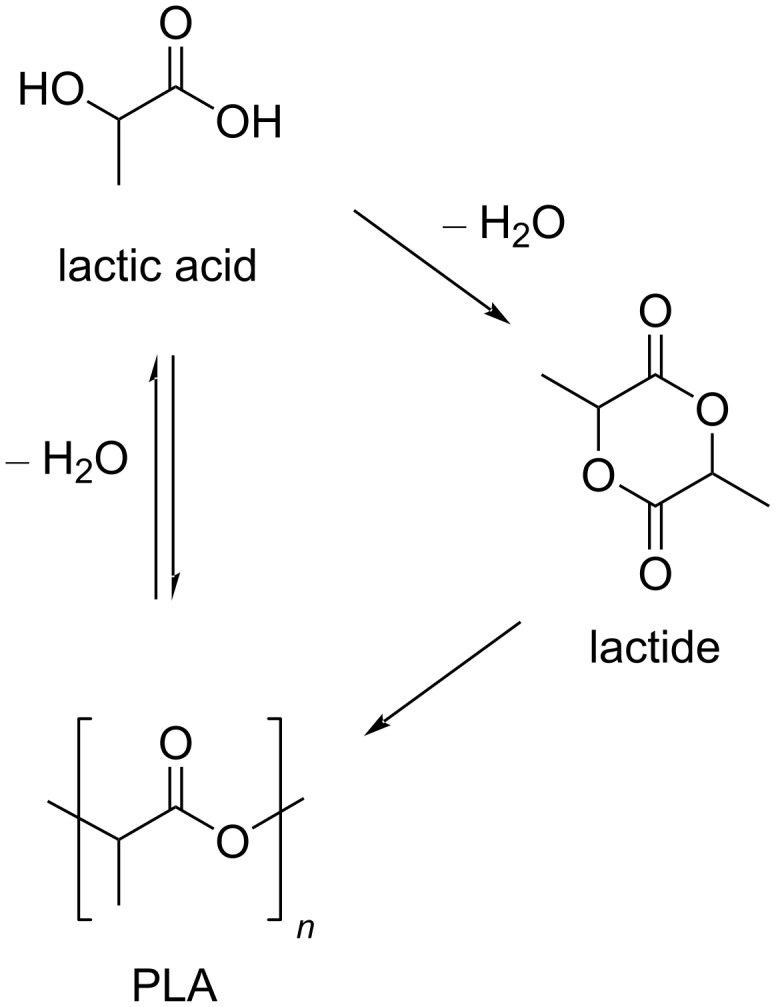
Synthetic routes to PLA.

**3.2.2 Polylactic acid (PLA):** PLA is a bioderived plastic [252] that is manufactured on a 190 kton scale directly from lactic acid by condensation or from lactide by ring-opening polymerisation (Scheme 8) [253–254]. The main renewable raw material for lactic acid is starch, e.g., from corn, cassava, sugarcane or beet pulp [255]. Owing to the chirality of lactic acid, three forms of PLA (ʟ, PLLA; ᴅ, PDLA; ᴅʟ, PDLLA) with slightly different properties (crystallinity, *T*_g_ 60–65 °C, *T*_m_ 130–180 °C) exist [256]. PLA is soluble in benzene, tetrahydrofuran, ethyl acetate, propylene carbonate and dioxane [257], and it is biodegradable [258–259]. Because of these features, PLA is largely employed in several applications: microelectronics, biomedicine and food packaging [260].

Although chemical recycling of PLA is possible by thermal methods over metal catalysts [261–262], these often result in a poor selectivity and in a variety of volatile compounds, with the notable exception of calcium oxide, which gave ʟ,ʟ-lactide from PLLA in ≈98% yield at 250 °C [263].

**Scheme 9:**
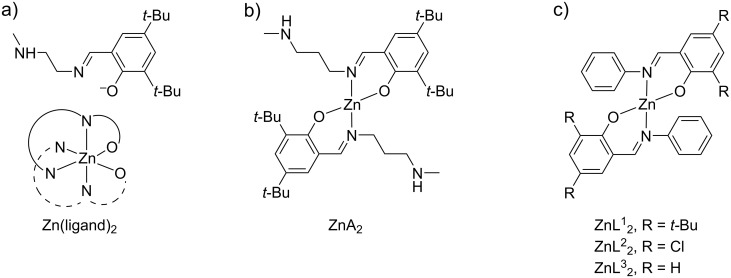
Structures of the zinc molecular catalysts used for PLA-methanolysis in various works. a) See [265], b) see [266], c) see [267].

**Scheme 10:**
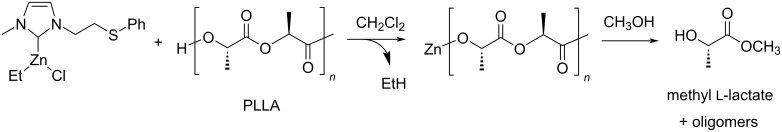
Depolymerisation of PLLA by Zn–N-heterocyclic carbene complex.

Alcoholysis. The solvolytic depolymerisation of PLA was mostly reported using zinc-based catalysts, ethanol or methanol agents, wherein a higher reactivity of the latter was ascribed to the better nucleophilicity. Indeed, a methyl lactate (Me-La) and ethyl lactate yield of 70% and 21% was obtained, respectively, using soluble zinc acetate at reflux temperature [264]. Interestingly, under the same reaction conditions, PET was unreactive, thus enabling the selectively recycle of mixed PET/PLA plastic waste. It was suggested that the different reactivity between PLA and PET is attributable to the amorphous, less rigid structure of PLA and to the potential of forming five-membered chetate ring intermediates between Zn(II) ions and lactate units, which favour the transesterification process. More recently, the use of soluble Zn(II) molecular catalysts was investigated. An in-depth study of the methanolysis reaction of PLA was carried out via design of experiments technique, using a series of imino monophenolate–Zn complexes and THF solvent. Different commercial samples of PLA were examined, showing the critical operational parameters to be temperature and catalyst concentration, whereas the process was not significantly affected by particle size or stirring speed. Thus, up to 100% Me-La yield was obtained using the six-coordinated Zn(ligand)_2_ complex sketched in Scheme 9a, either at 90 °C and 16 wt % catalyst or 130 °C and 8 wt % catalyst [265]. Higher efficiency was provided by the tetrahedral complex ZnA_2_ bearing a similar ligand, as shown in (Scheme 9b), which resulted in 81% Me-La selectivity at full PLA conversion at under 50 °C and 4 wt % catalyst loading [266]. However, in the absence of THF solvent, the latter catalysts gave a 98% Me-La yield at 130 °C. The comparable complexes ZnL^1^_2_, ZnL^2^_2_ and ZnL^3^_2_ shown in Scheme 9c resulted in a lower catalytic efficiency (Me-La yield 41–88%, 50 °C, 8 wt % catalyst loading, 18 h), and thus indicating a significant steric and electronic effect of the ligand [267]. A reaction mechanism for PLA depolymerisation was proposed, consisting of two consecutive first-order steps, in which Me-La production follows the formation of chain-end groups intermediates [265]. A zinc–N-heterocyclic carbene complex was used as catalysts for the methanolysis reaction of PLLA via a two-step, one-pot procedure using CH_2_Cl_2_ solvent and an excess of methanol at room temperature (Scheme 10) [268]. At full substrate conversion, a Me-La/oligomers ratio around 10:1 was detected by GPC analysis. Notably, mutatis mutandis, almost all of the above described zinc complexes could be used as catalysts, both in PLA alcoholysis and in PLA synthesis via lactide polymerisation.

**Scheme 11:**
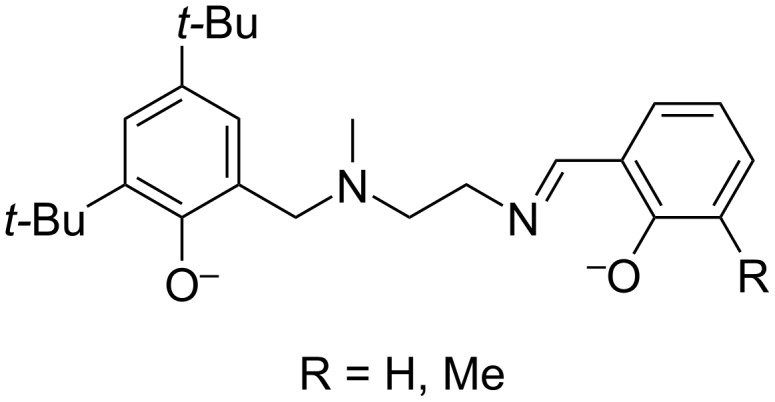
Salalen ligands.

Metal species other than zinc were reported as effective catalysts for PLA methanolysis. Group 4 metal complexes with salalen ligands of the formula M(ligand)(OiPr)_2_ (M = Ti, Zr, Hf) were used in the methanolysis reaction of PLA at room temperature with an excess of methanol and CH_2_Cl_2_ cosolvent (Scheme 11) [269–270]. A 75% yield of Me-La and residual oligomers with *M*_n_ 500 g⋅mol^−1^ were obtained by conversion of *M*_n_ 200000 g⋅mol^−1^ PLA using the hafnium derivative. As an alternative to expensive metal (complex) catalysts, methanolysis of PLA was recently described using alkali metal halides [271]. In an optimised experiment, PLA from various goods (cups, fork, spoons, containers, *M*_w_ 120000–260000 g⋅mol^−1^) was converted into Me-La in up to 97% yield using 2.5 mol % KF, 180 °C microwave heating and 23.1 equiv CH_3_OH. The potassium fluoride catalyst could be reused in up to three runs with no change in performance, while a 50% drop of the yield was observed afterwards.

Me-La is a low-toxic chemical used as substitute for hydrocarbon solvents, with applications in the field of paints, lacquers and cleaning agents [272–273]. It should be finally mentioned that during the alcoholysis reaction of PLA using alkoxide catalysts, alkaline earth metal adducts (typically of Ca^2+^) were isolated, and thus suggesting a potential involvement of the metal centre in the depolymerisation mechanism [274–275].

**Scheme 12:**
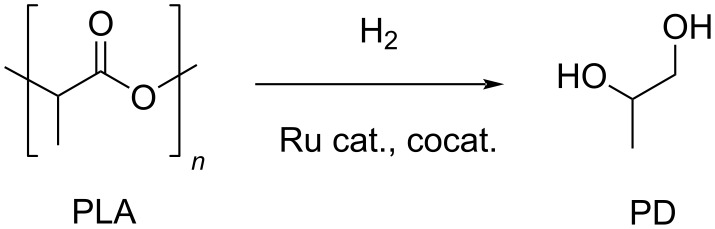
Catalytic hydrogenolysis of PLA.

Hydrogenolysis. The Ru(triphos)tmm/HNTf_2_ catalytic system described above for the hydrogenolysis reaction of PET was also successfully applied in PLA hydrogenative depolymerisation (Scheme 12) [191]. PLA was directly converted to 1,2-propanediol in 99% yield using 1 mol % catalyst (with respect to lactic acid units) and 1,4-dioxane solvent at 140 °C and 100 bar H_2_. A TOF of 6.19 mol_PD_⋅mol_Ru_^−1^⋅h^−1^ can be calculated based on this. Scale-up using PLA granulates and beverage cups was also possible using a lower catalyst loading. In addition, the method allowed for the selective recycle of equimolar mixtures of PET and PLA using the [Ru(triphos-xyl)methylallyl]NTf_2_ catalyst congener at 45 °C reaction temperature, wherein insoluble PET was filtered out, while PLA was fully converted to PD. Similarly to PET, the ruthenium(II)–PNN complex sketched in Table 1, entry 2 was also used in PLA hydrogenolysis to give PD in 99% yield at 160 °C, 54 bar H_2_ and in anisole/THF solvent [182,188]. PD is a safe chemical that is mainly produced from propylene oxide or catalytically from lactic acid intermediate, and it serves in the polymer and food industry or as antifreezing agent [276–277].

**Scheme 13:**
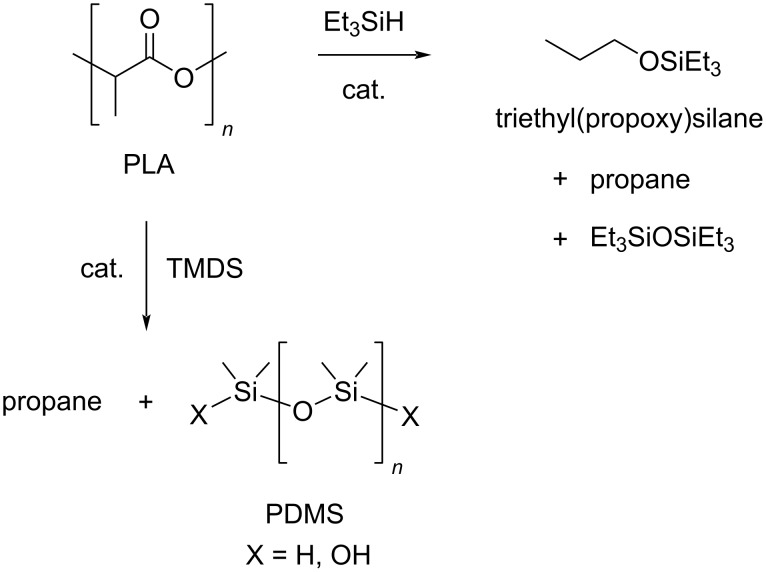
Catalytic hydrosilylation of PLA.

Under milder reaction conditions, PLA could be converted to the corresponding silyl ether in 92% yield, propane and silicon byproducts (8%) using the above mentioned Brookhart pincer complex [Ir(POCOP)H(THF)][B(C_6_F_5_)_4_] shown in Scheme 3, an excess of Et_3_SiH and chlorobenzene solvent at 90 °C (Scheme 13) [193]. The use of 6 equiv of 1,1,3,3-tetramethyldisiloxane (TMDS) led to the total conversion to propane and polydimethylsiloxane (PDMS), a silicon oil with several applications (lubricants, food-additives, breast implants).

**Scheme 14:**
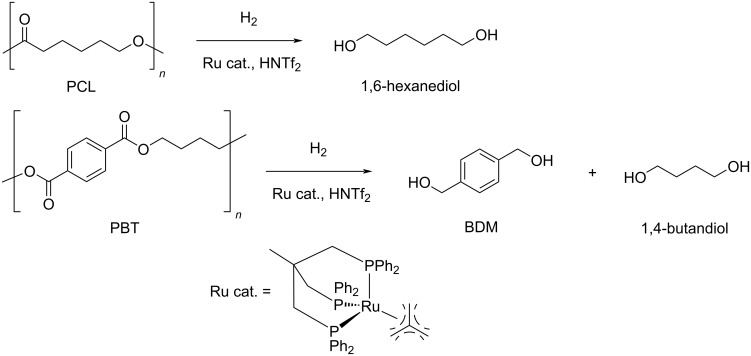
Hydrogenative depolymerisation of PBT and PCL by molecular Ru catalysts.

**3.2.3 Other polyesters:** The hydrogenolysis reaction of esters other than PET and PLA was carried out using the above described soluble Ru(triphos)tmm/HNTf_2_ catalytic system [191]. Thus, poly(butylene terephthalate) (PBT) and polycaprolactone (PCL) were depolymerised into the corresponding (co)monomeric diols at 140 °C and 100 bar H_2_ in 1,4-dioxane solvent (Scheme 14). A 99% selectivity to 1,6-hexanediol was observed at full PCL conversion, whereas the selectivity to diols was only 22% for PBT (due to the formation of ethers byproducts), which could be raised to 99% by using the bulkier Ru(triphos-xyl)tmm catalyst derivative.

PCL could be converted to 1,6-hexanediol in 68% yield also through a two-step procedure involving hydrosilylation by the above mentioned cationic Ir catalyst complex [Ir(POCOP)H(THF)][B(C_6_F_5_)_4_] and TMDS reagent (chlorobenzene solvent, 90 °C), followed by alkaline hydrolysis (10% NaOH in CH_3_OH/H_2_O) [193]. PCL is a biodegradable polymer with a low melting point (≈60 °C) and glass transition temperature (−60 °C). It is commonly used in the manufacture of polyurethanes, to which it imparts improved solvent resistance, flexibility and toughness [278].

**Scheme 15:**
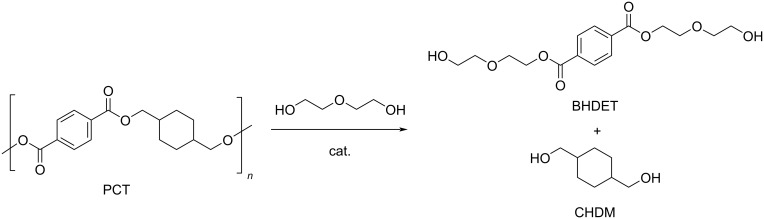
Glycolysis reaction of PCT by diethylene glycol.

The glycolysis of poly(1,4-cyclohexylenedimethylene terephthalate) (PCT) was reported using DEG as reagent and zinc acetate as catalyst (0.12 mol %, Scheme 15) [279]. A 56% yield of bis(2-hydroxydiethylene terephthalate) (BHDET) was obtained at 196 °C and a DEG/PCT 15:1, w/w ratio, which was five times lower than that using PET under the same reaction conditions. This finding was attributed to the steric hindrance of the 1,4-cyclohexanedimethanol (CHDM)-based chain that hampers the transesterification process. A 90% BHDET yield was achieved using Zn(OCH_3_)_2_ catalysts under the same conditions.

**Scheme 16:**
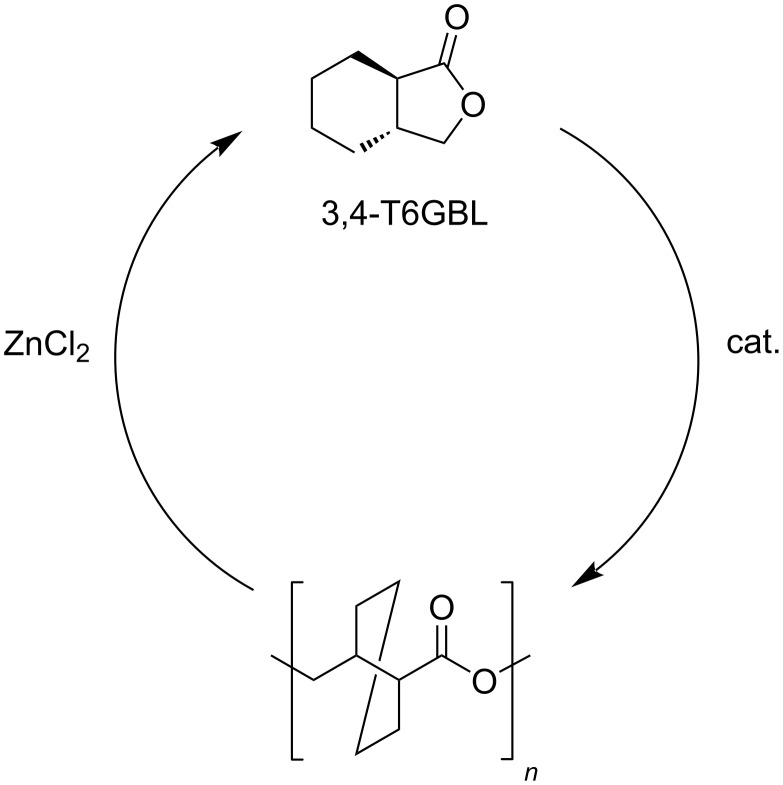
Polymerisation–depolymerisation cycle of 3,4-T6GBL.

Recently, a quantitatively and selectively depolymerisable novel polyester was developed based on a *trans*-fused six-membered γ-butyrolactone ring, 3,4-T6GBL (Scheme 16) [280]. This material joins the advantages of the ease of depolymerisation (97% monomer yield, 180 °C, toluene, 2 mol % ZnCl_2_ catalyst), rigid structure of the monomer (which provides the polymer with good thermal and mechanical properties) and facile synthesis (ring-opening polymerisation, solvent-free, room temperature, La, Y or Zn catalyst), significantly contributing to a closed-loop concept of plastics recycle. The approach enabled to perform the polymerisation–depolymerisation cycle over three times and on a multigram scale, using both linear and cyclic polymers. These advantages are not provided, for example, by more conventional poly(γ-butyrolactone) plastics, which require high depolymerisation temperatures (260–300 °C) and undesirable synthetic conditions (−40 °C) [281].

**Scheme 17:**
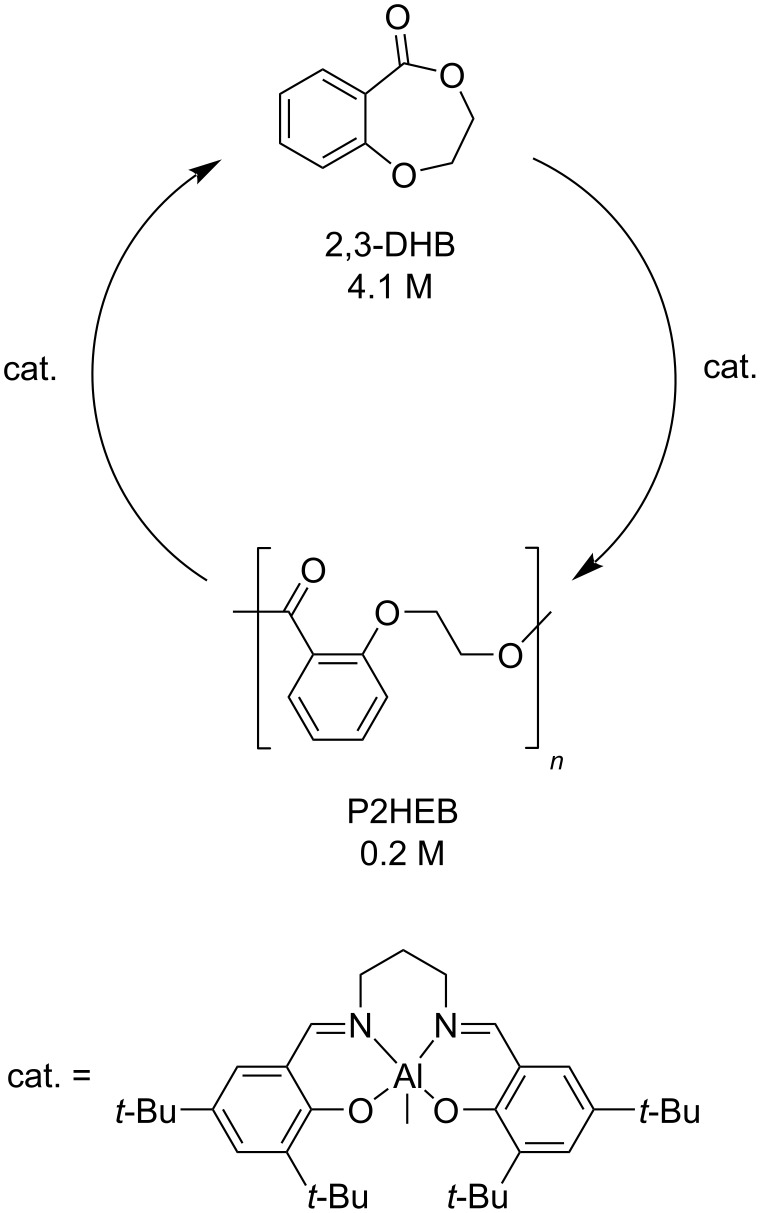
Polymerisation–depolymerisation cycle of 2,3-HDB.

Following the same approach to purposely designed, chemically recyclable polymers, it was reported that poly(2-(2-hydroxyethoxybenzoate) (P2HEB) is reversibly depolymerised to 2,3-dihydro-5*H*-1,4-benzodioxepin-5-one (2,3-DHB) in 94% yield by an aluminium–salen catalyst at room temperature (Scheme 17) [282]. Thus, a polymerisation–depolymerisation cycle could be established using the same metal catalyst, simply by adjusting the initial monomer concentration in a one-pot process.

#### 3.3 Polycarbonates

**3.3.1 Poly(bisphenol A carbonate) (PBPAC):** Bisphenol A (BPA) is a monomer for a variety of polymers widespread in our everyday life, namely polycarbonates, polyesters, polyethers, polysulphones and epoxy resins [249,283]. Particularly, PBPAC is used in the manufacture of plastics for food and beverage containers, safety helmets, optical lenses, electronic and medical equipment, phones, automotive components and toys [284–285]. This justifies for BPA to be one of the highest-volume chemicals produced worldwide, with a global market of around 6 million tons in 2017, 68% of which account for the manufacture of polycarbonates [286–287]. However, BPA is considered a hazardous substance [288–289] and an endocrine disrupting agent [290–291]. BPA can leach from the corresponding polymers, including water- and temperature-sensitive polycarbonates [292–293]. BPA is industrially obtained by the condensation reaction of phenol with acetone, using an excess of phenol [294]. All byproducts of the process, including unreacted phenol, are toxic [295–296], whereas a purity greater than 98% is required for most BPA applications [297–298]. PBPAC is then produced by the condensation of BPA and a carbonyl source, usually phosgene or diphenyl carbonate [299–300]. Commercial PBPAC is a tough material with an average *M*_w_ of 50000 g⋅mol^−1^ and *T*_g_ around 150 °C. It is soluble in THF, hazardous NMP and chlorinated solvents and insoluble in alcohols and water [301]. A number of chemolytic processes have been developed in the recent years for the selective depolymerisation of PBPAC, including hydrogenolysis, hydrolysis, alcoholysis and aminolysis, some of which are metal-catalysed [302–303].

**Scheme 18:**
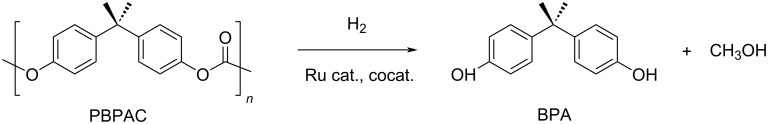
Hydrogenative depolymerisation of PBPAC by molecular Ru catalysts.

Hydrogenolysis. The hydrogenative depolymerisation of PBPAC was accomplished through the Ru–triphos molecular catalyst described above for PET, PLA, PBT and PCL polyesters [191]. Thus, use of the soluble Ru(triphos)tmm complex, in conjunction with acid HNTf_2_ cocatalyst (1:1) in 1,4-dioxane, resulted in complete conversion and selectivity to BPA and methanol under the moderate conditions of using 100 bar H_2_ at 140 °C (Scheme 18 and Table 5, entry 1). The protocol could be successfully extended to the depolymerisation of consumer goods, such as compact discs and beverage cups. Notably, pure BPA could be recovered in >70% yield after simple CH_2_Cl_2_ extraction. A similar approach was recently reported using commercially available Ru(II) catalysts, namely the Milstein [304] and the Ru–MACHO–BH [305] complexes bearing tridentate PN ligands, as shown in Table 5, entries 2 and 3, which are known as efficient hydrogenation catalysts of organic carbonates [306–307]. High BPA yields were obtained in those experiments as well, though with lower hydrogen pressure and catalyst productivity in terms of turnover frequency (mol_BPA_⋅mol_Ru cat._^−1^⋅h^−1^). Potassium *tert*-butoxide was used as cocatalyst, the role of which was speculated to activate the carbonate group of the polymer [308]. The depolymerisation of a digital versatile disc (DVD) using the latter catalyst afforded BPA in an estimated 97% yield after THF pretreatment.

**Table 5:** Hydrogenolysis of PBPAC by soluble Ru molecular catalysts.

entry	catalyst	cocatalyst^a^	reaction conditions^b^			conv.^c^ (%)	BPA		reference
	
			*T* (°C)	H_2_ (bar)	solvent		sel.^d^ (%)	TOF^e^ (mol_BPA_⋅mol_cat._^−1^⋅h^−1^)	

1	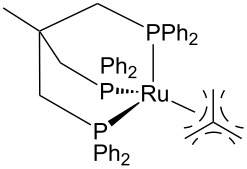	HNTf_2_^f^	140	100	1,4-dioxane	99	99	6.12^g^	[191]
2	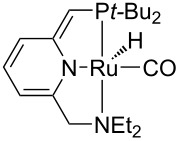	K*t-*BuO	140	45	THF	99	99	1.10	[304]
3	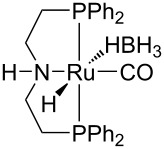	K*t-*BuO	140	45	THF	99	99	0.06	[305]

^a^Catalyst loading 5 mol %, calculated based on PBPAC repetition units, cocatalyst molar ratio = 1:1. ^b^Reaction temperature, hydrogen pressure, 24 h reaction time. ^c^PBPAC conversion. ^d^Selectivity to BPA. ^e^Turnover frequency, calculated based on PBPAC repetition units and moles of Ru catalyst. ^f^Catalyst loading 1 mol %. ^g^Reaction time 16 h.

**Scheme 19:**
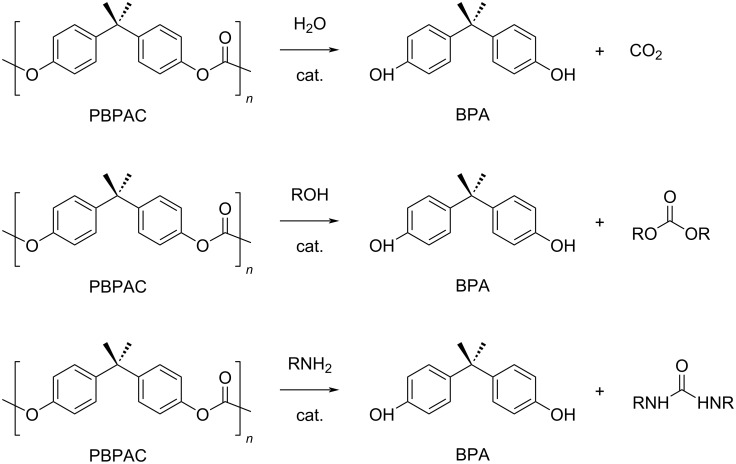
Catalytic hydrolysis (top), alcoholysis (middle) and aminolysis (bottom) reactions of PBPAC.

Hydrolysis. The hydrolytic depolymerisation of PBPAC in hot compressed water was achieved via manganese acetate catalyst (Scheme 19, top) [309]. Under optimal conditions (280 °C, catalyst loading 2 wt %), the reaction resulted in 55% selectivity to BPA and 19% to phenol at full polymer conversion. A higher selectivity to BPA was obtained by simple Lewis acid treatment using rare earth metal triflate catalysts [310]. The process occurred in the homogeneous phase using a THF/H_2_O solvent mixture and a H_2_O/PBPAC weight ratio of ≤1. The highest BPA yield (97% at 160 °C) was observed for La(CF_3_SO_3_)_3_ due to the reduced decomposition of BPA to phenol, 4-isopropenylphenol and 4-isopropylphenol. A comparison with triflic acid catalyst ruled out the possibility of a proton-catalysed depolymerisation process.

Alcoholysis. A variety of metal-based catalytic systems was recently described for the alcoholysis reaction of PBPAC using diverse alcohols (Scheme 19, middle). Thus, a Lewis acid catalyst consisting of a soluble FeCl_3_–ionic liquid adduct, namely BmimCl·2FeCl_3_, was reported for the methanolysis of PBPAC, providing BPA in 97% yield at 120 °C [311]. Higher alcohols resulted in lower BPA yields. A mechanism was hypothesised in which the iron centre activates the carbonyl group of the polymer toward nucleophilic attack of methanol. The catalyst could be efficiently recovered and reused over six runs, after ethyl acetate/water extraction. On the other hand, CeO_2_–CaO particles onto hollow ZrO_2_ nanospheres were used as heterogeneous catalyst for the methanolysis of PBPAC, yielding around 95% BPA at 100 °C in a THF/methanol mixture [312]. The basic sites, due to lattice O^2−^ of CeO_2_, were attributed to be responsible for the deprotonation of methanol, and thus initiating the solvolytic process in that reaction. Depolymerisation of PBPAC was reported using various alcohols (methanol, phenol, benzyl alcohol, 1-naphthol, PD, glycerol), a mechanical mixture of zinc oxide NPs and tetrabutylammonium chloride as catalyst as well as THF cosolvent to give BPA and the corresponding carbonates in >98% yield at 100 °C reaction temperature (Scheme 19) [313]. The insoluble Lewis acid ZnO catalyst could be removed from the reaction mixture by centrifugation and reused five times with a minor loss of activity. However, Bu_4_NCl was only partially recovered and had to be integrated with fresh cocatalyst after each run. Remarkably, the reaction with glycerol enabled the recycling of two industrial wastes (PBPAC and glycerol) into the valuable chemicals BPA and glycerol carbonate in one process only, with the latter compound being industrially used as synthetic intermediate, solvent and in the formulation of cosmetics [314].

Aminolysis. The ZnO–Bu_4_NCl Lewis acid catalytic mixture was also successfully used in the aminolytic depolymerisation of PBPAC by different amines (cyclohexylamine, aniline, imidazole, 1,2-diaminopropane, 1,3-diaminopropane) to give the corresponding substituted (cyclic) ureas in >97% yield (Scheme 19, bottom) [313]. The reaction with 2-amino-1-propanol gave 4-methyloxazolidin-2-one. Despite the complications due to separation from BPA, the process provides ureas of industrial relevance as anticancer or antiviral agents [315–316].

**3.3.2 Poly(propylene carbonate) (PPC) and poly(ethylene carbonate) (PEC):** PPC is a thermoplastic material obtained by the copolymerisation of CO_2_ with propylene oxide or propylenediol, which is mainly used as a packing material and in binder applications [317]. It has a low thermal stability, with a decomposition temperature around 200 °C and a *T*_g_ around 40 °C, depending on the molecular weight. PPC may be readily dissolved in many solvents (e.g., chlorinated hydrocarbons, THF, benzene, ethyl acetate and lower ketones), but it is insoluble in longer-chain alkanes, alcohols and water [318].

**Scheme 20:**
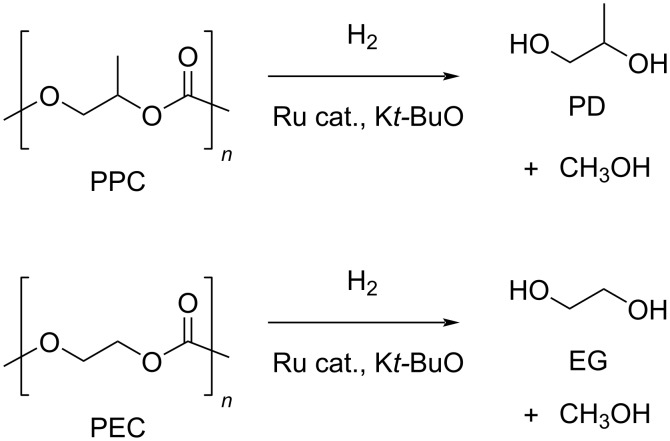
Hydrogenative depolymerisation of PPC (top) and PEC (bottom) by molecular Ru catalysts.

Hydrogenative depolymerisation of PPC and PEC to methanol and the respective glycols (PD and EG, respectively) was achieved using the soluble Milstein ruthenium catalysts described above for the hydrogenolysis of PET (Scheme 20) [188]. Thus, more than 91% glycol yield was obtained using a 1:2 Ru catalyst/butoxide molar ratio, 160 °C reaction temperature, 55 bar H_2_ pressure and an anisole/THF 1:1 solvent mixture (Table 6, entries 1 and 2). The same approach was adopted using a Ru(II)–PNP pincer complex, showing higher catalytic activity (TOF 41.3 h^−1^) under similar reaction conditions (Table 6, entry 3) [319]. The role of butoxide was proposed to be the conversion of the staring molecular complex in the catalytically active species by elimination of HCl. Similarly, a nonprecious PNN–manganese carbonyl complex was reported to afford PD from PPC in 91% yield (Table 6, entry 4) [320]. By contrast, use of a comparable Mn complex and KH as activator resulted in a much lower selectivity to PD (68%) at full PPC conversion (110 °C, 50 bar H_2_, in toluene), resulting in the formation of a propylene carbonate byproduct [321].

**Table 6:** Hydrogenolysis of PPC by molecular metal catalysts.

entry	catalyst	cocatalyst^a^	reaction conditions^b^			conv.^c^ (%)	PD		reference
	
			*T* (°C)	H_2_ (bar)	solvent		sel.^d^ (%)	TOF^e^ (mol_PD_⋅mol_cat._^−1^⋅h^−1^)	

1	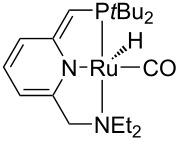	K*t-*BuO	160	55	anisole/THF^f^	99	100	4.12	[188]
2	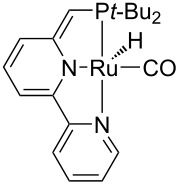	K*t-*BuO	160	55	anisole/THF^f^	99	100	4.12	[188]
3	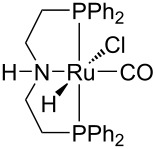	K*t-*BuO^g^	140	50	THF	99	99	41.25	[319]
4	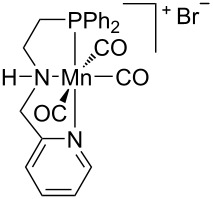	K*t-*BuO^h^	140	50	1,4-dioxane	91	100	2.84^i^	[320]
5	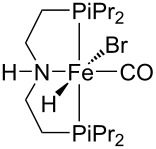	K*t-*BuO^j^	140	—	iPrOH/THF^k^	65	100	0.43	[322]

^a^Catalyst loading 1 mol %, calculated based on PPC repetition units, cocatalyst molar ratio = 2:1. ^b^Reaction temperature, hydrogen pressure, 24 h reaction time. ^c^PPC conversion. ^d^Selectivity to PD. ^e^Turnover frequency, calculated based on PPC repetition units and moles of Ru catalyst. ^f^1:1, v/v. ^g^Catalyst loading 0.1 mol %, cocatalyst molar ratio = 1:1. ^h^Catalyst loading 0.2 mol %, cocatalyst molar ratio = 2:1. ^i^Reaction time 16 h. ^j^Catalytic hydrogen transfer. Catalyst loading 5 mol %, cocatalyst molar ratio = 1:1, reaction time 30 h. ^k^4:1, v/v.

In the search of safer and “greener” alternatives, a slightly different approach to controlled PPC depolymerisation was recently proposed, based on catalytic hydrogen transfer rather than hydrogenation reaction, and thus to avoid involvement of high H_2_ pressures [322]. Thus, hydrogen transfer from isopropanol to PPC using a soluble iron pincer-type catalyst resulted in a 65% PD yield at 140 °C (Table 6, entry 5). However, a relatively high amount of catalyst was required. Potassium butoxide and THF were used as precatalyst activator and cosolvent, respectively.

**Scheme 21:**
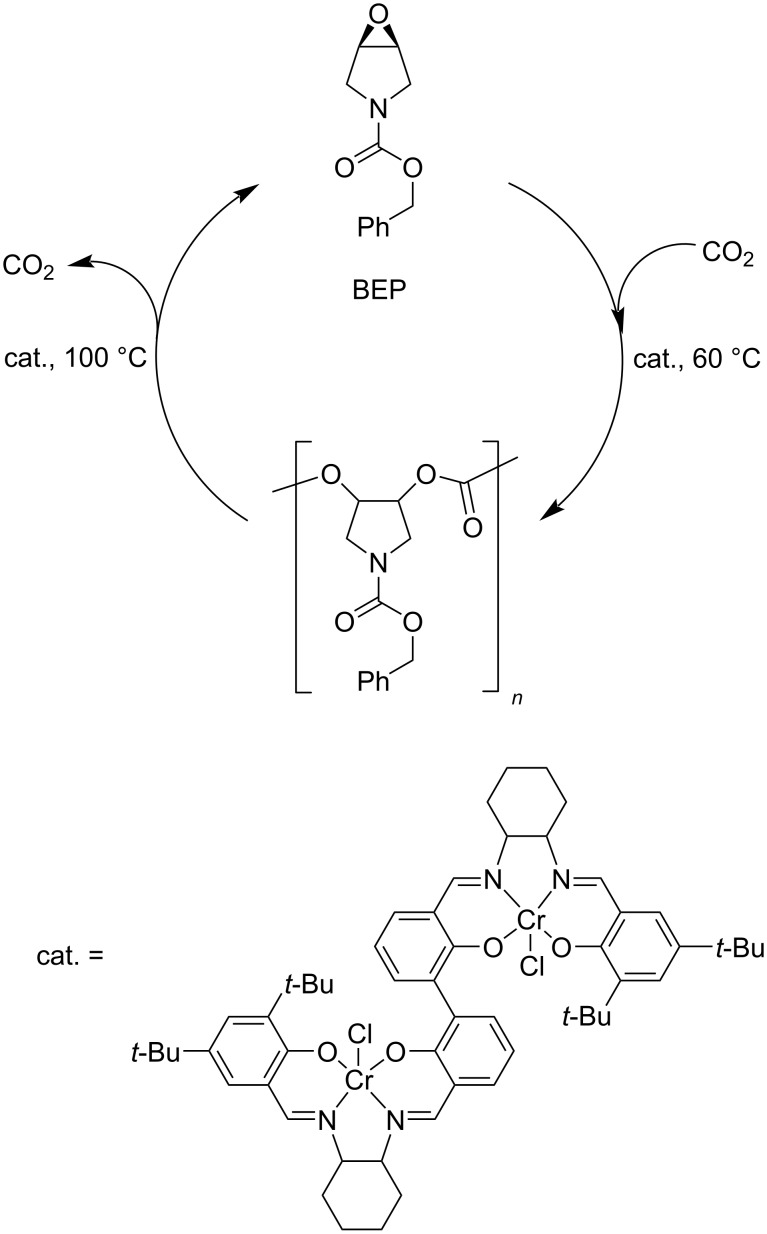
Polymerisation-depolymerisation cycle of BEP.

**3.3.3 Other carbonates:** A polycarbonate suitable for smooth chemical recycle was engineered based on 1-benzyloxycarbonyl-3,4-epoxypyrrolidine (BEP) units [323]. In fact, a one-pot copolymerisation–depolymerisation cycle was enabled using a dinuclear salen–chromium complex in the presence of a bis(triphenylphosphine)iminium cocatalyst (Scheme 21). Therein, the BEP monomer was fully converted to the polycarbonate at 60 °C reaction temperature, while complete and selective depolymerisation back to BEP was achieved at 100 °C. The process could be repeated several times with no changes in either the monomer or the copolymer structure. After removing the catalyst, the polymer was stable at 200 °C for 10 h.

#### 3.4 Polyamides (PA)

**Scheme 22:**
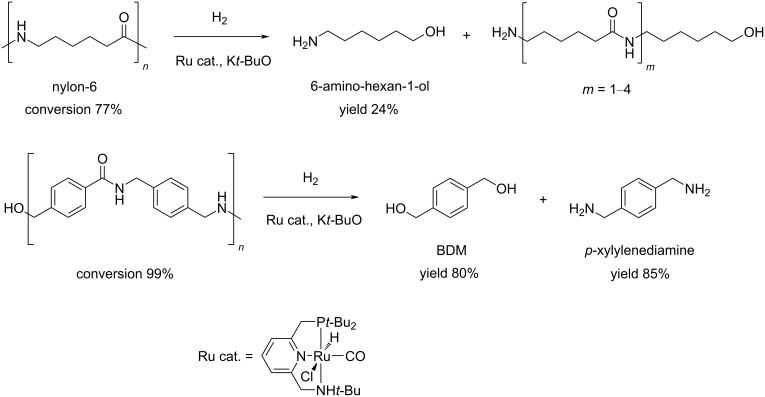
Hydrogenolysis of polyamides using soluble Ru catalysts.

Polyamides may be natural (e.g., silk, wool) or synthetic polymers (e.g., nylons, aramids, polyaspartates) that are largely used in the manufacture of fibres and automotive components as well as in biomedicine, due to their excellent mechanical and thermal properties [324–325]. The widespread use of polyamides and the high price of the starting monomers, such as ε-caprolactam, have led to the development of several methods for their chemical recycling. Most of these are based on mineral acid [326], organic base (e.g., 4-dimethylaminopyridine, triethylenetetramine) [327–328] or organic acid (e.g., glycolic, lactic, benzoic acid) [329] catalysis, using supercritical methanol or ionic liquids as solvent [330]. Few literature reports exist on the depolymerisation of polyamides using metal catalysts. In an earlier paper, the hydrolysis of nylon-6 was achieved by a combination of zinc chloride (40 wt %) and phosphoric acid (20 wt %) under microwave irradiation, however, resulting in a mixture of linear and cyclic oligomers at 89% polymer conversion [121,331]. While drafting the present review, the first example of catalytic hydrogenative depolymerisation of polyamides and polyurethanes was described, using soluble Milstein-type Ru–pincer complexes (2 mol %), DMSO solvent and K*t-*BuO cocatalyst at 150 °C and 70 bar H_2_ [332]. Typically, a selectivity to the corresponding diols/diamines/amino alcohols in the range of 20–80% was observed at 60–99% conversion, depending on the polymeric substrate. For instance, 6-amino-hexan-1-ol and BDM were obtained in 24% and 80% yield, respectively, from nylon-6 and the polyamide shown in Scheme 22.

#### 3.5 Other plastics

**Scheme 23:**
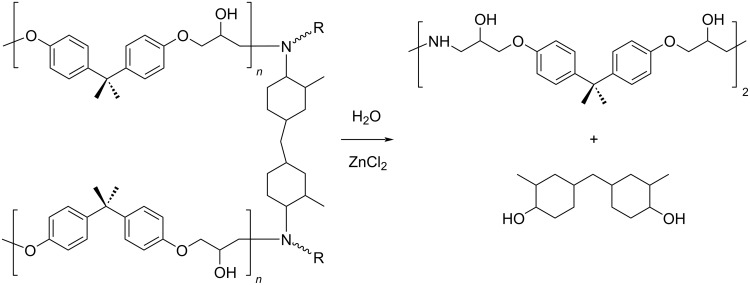
Catalytic depolymerisation of epoxy resin/carbon fibres composite.

**3.5.1 Epoxy resins (EP):** EP are thermosetting polymers featuring high thermal and chemical resistance. They are widely used in the manufacture of paints, metal coatings, electronic components and adhesives [333]. EP are usually reinforced with fibres to give composite materials for the aeronautical, automotive and sport industries. Actually, recycling efforts of EP were mainly focused on the recovery of valuable (expensive) carbon fibres rather than the polymers themselves. Recently, a metal-catalysed route was reported for the degradation of the epoxy resin of bisphenol A diglycidyl ether (BADGE)–carbon fibres composites [334]. Therein, low-coordinated aquo ions of zinc enabled the selective cleavage of the R_2_CH–N bond while keeping intact RCH_2_–N, C–C and C–O bonds (Scheme 23). The method was previously adopted for the conversion of cellulose to hydroxymethylfurfural and required the use of highly soluble zinc chloride to obtain a concentrated aqueous solution of metal (60 wt % ZnCl_2_) [335]. On this basis, the small, incompletely coordinated Zn^2+^ ions were proposed to activate the selective cleavage of C–N bonds, acting as Lewis acid centres. The process carried out at 220 °C led to carbon fibres, a dimer of DGEBA reused for the synthesis of new EP, and 4,4'-methylenebis(2-methylcyclohexanol). The concentrated ZnCl_2_ solution showed good reusability, and thus adding some advantages to common highly energy-consuming methods.

**3.5.2 Polyethers:** Polyethers are polymers with a solubility that depends heavily on the solvent used, including water, and they find applications in the cosmetic, pharmacy or paint industries [336]. Thermal decomposition or disposal into landfills are consolidated management systems of “end-of-life” polyethers [337–338], whereas very few studies cope with the catalytic depolymerisation through selective C–O bond cleavage into specific low-molecular-weight chemicals.

**Scheme 24:**
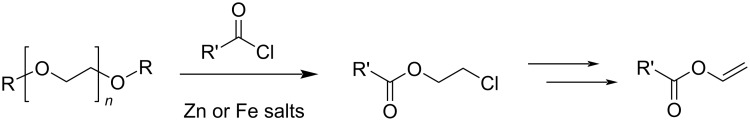
Depolymerisation of polyethers with metal salt catalysts and acyl chlorides.

**Scheme 25:**
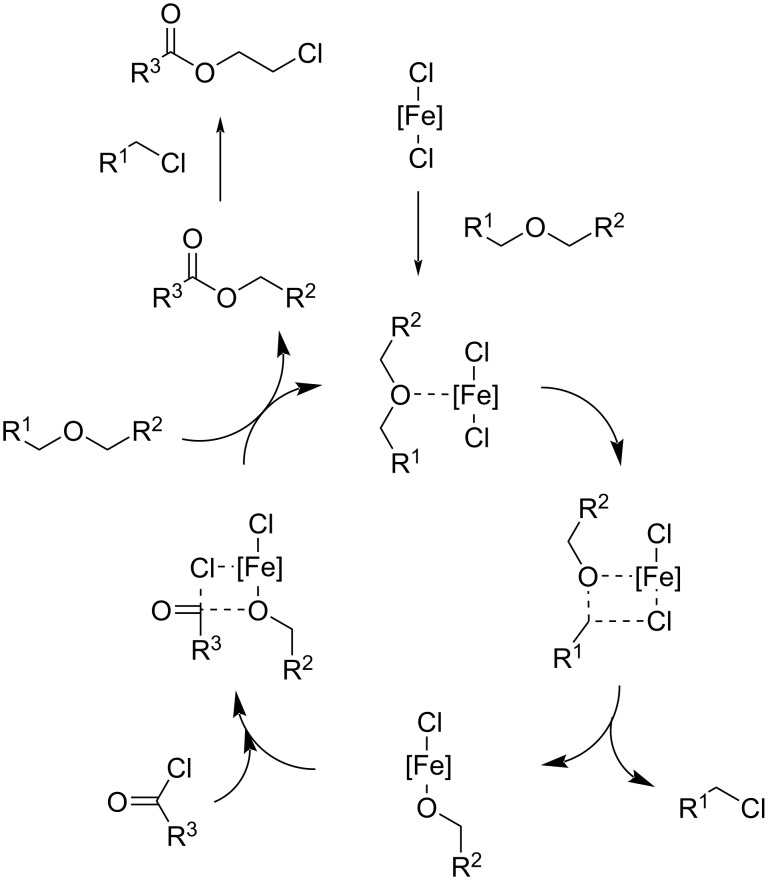
Proposed mechanism for the iron-catalysed depolymerisation reaction of polyethers. Adapted with permission from [341], Copyright © 2012 WILEY-VCH Verlag GmbH & Co. KGaA, Weinheim. Used with permission from Stephan Enthaler and Maik Weidauer, “Low‐Temperature Iron‐Catalysed Depolymerization of Polyethers”, ChemSusChem, John Wiley and Sons.

The group of Enthaler reported a number of research with a common strategy for polyethers depolymerisation [339–341]: Basically, the solvent-free reaction of a polyether with an acyl chloride in the presence of a catalytic amount (5 mol %) of zinc or iron salts as Lewis acid catalysts results in monomeric chloroesters, which are valuable chemicals reprocessable into other polymers (Scheme 24). A deep study was carried out investigating the effect of key reaction parameters: metal salt, catalyst loading, temperature, depolymerisation agent and the applicability to a variety of polyethers. Successful examples include depolymerisation of polyethylene glycol (PEG) and polytetrahydrofuran (polyTHF) to chloroesters in 70–78% and 92% yield, respectively, using ZnCl_2_ at 130 °C or Zn(OTf)_2_ catalyst when acetic anhydride was used as depolymerising agent [339–340]. Chloroester yields in the range 89–95% were obtained for PEG depolymerisation at 100 °C using FeCl_2_ as catalyst [341]. A mechanism was postulated in which the ether bond is cleaved via formation of an iron alkoxide intermediate (Scheme 25). Sustainability issues relate to the hazardous properties of low-molecular-weight acyl chlorides, which could be partially circumvented by the use of bioderived fatty acid chlorides [340].

In a different approach, selective ring-closing depolymerisation of polyTHF to THF was achieved in 92% and 95% yield, respectively, using Lewis acid catalysis by FeCl_3_ (5 mol %, 180 °C) [342] or phosphotungstic acid (10 wt %, 130 °C) [343].

## Conclusion

The implementation of a value chain for plastics, in which design is coupled with production, use, depolymerisation and reprocessing, may substantially contribute to the development of a truly circular economy model for plastic materials, wherein scraps are converted into useful chemicals and reusable building blocks [344]. This model addresses three grand challenges of our century: pollution of the habitat, carbon dioxide emissions and dependence on fossil sources. However, performing plastics recycling via chemical processing is not enough. This must be achieved at competitive economic and environmental costs [345]. A perusal of the recent literature indicates that, despite their urgency and significance, strategies for sustainable chemical recycling are surprisingly still rather underdeveloped. Most methods for controlled and selective depolymerisation rely on harsh reaction conditions, use of an excess of reagents, toxic solvents and troublesome downstream treatments often generating a considerable amount of waste. Metal catalysis may represent a useful tool to overcome these issues, provided that significant improvements are achieved in relation to key challenges.

### Catalysts

Most processes, particularly if based on solvolysis reactions, are carried out using traditional metal salt-based catalysts (e.g., zinc chloride, zinc acetate), the role of which is merely to act as Lewis acid centres, and they are often needed in a large amount with respect to the plastic to be depolymerised (i.e., >2 and up to 60 wt %). In some cases, toxic metals (e.g., Co, Ni) result in the highest catalytic efficiency. A significant improvement is represented by the use of molecular complex catalysts, particularly based on ruthenium for use in hydrogenolysis. These systems, typically used in up to 2 wt %, are quite efficient under relatively mild reaction conditions, although a strong acid or basic cocatalyst is usually required. On the other hand, soluble catalysts were mainly investigated so far, with clear limitations in terms of catalyst separation and reuse, purification, scale-up and cost. The use of meal-based ionic liquids and deep eutectic solvents has also been explored; however, their viability for practical application at large is uncertain [346]. Thus, inventive catalysts shall be developed with particular attention to heterogeneous systems, for instance, solid-supported metal species based on low-cost metals and solid materials featured by enhanced mass transfer and thermal resistance properties. Bifunctional solid catalysts bearing arrays of metal and acid single-sites may be useful in the same instances and processes [347–348].

### Polymers

Easily and selectively depolymerisable plastics are relatively uncommon, owing to the poor mechanical and physical properties and to the low temperature required to achieve their polymerisation [280]. Specifically designed, chemically recyclable polymers have been developed in some cases, which may offer an affordable solution to this regard (see, e.g., Section 3.2.3). These materials are indeed usable in closed polymerisation–depolymerisation loops, and hence appropriate to extend the life cycle of plastics. Excellent reviews describe the recent developments in the field [71].

### Processes

Large-scale chemical recycling of plastics is thus far hampered by the higher costs compared to mechanical recycling. A reason for this lays in the scarce development of effective catalysts. On the other hand, most processes for chemical recycling are still based on conventional organic reactions, requiring an excess of (unstable) decomposing agents or high temperature (e.g., for transesterifications). Moreover, performing the reactions in the homogeneous phase improves their kinetics, which, however, is limited by the usual poor solubility of polymers, and thus often ending up in the use of toxic solvents (e.g., chlorinated ones). Hence, besides the need for more efficient catalysts, the use of renewable and safer reagents and solvents is certainly desirable [349–350]. More importantly, novel processes and depolymerisation strategies have to be designed. Whereas glycolysis and methanolysis methods have reached commercial maturity (e.g., for PET), at high energy costs, however, in other instances (e.g., for polyolefins), no efficient processes for selective depolymerisation are in place yet. Hydrogenative depolymerisation represents a promising contribution to this end, owing to the use of the clean reducing agent H_2_, no need for organic reactants, reduced amount of metal catalyst, milder reaction conditions and limited formation of byproducts. Issues related to hydrogen supply may be circumvented, e.g., by in situ-generated hydrogen. Novel processes have thus to be developed, with special emphasis on those based on reaction sequences in one-pot or depolymerisation–polymerisation cycles as they clearly benefit from reduced energy inputs, reactor volumes and units, no need for intermediate purification/separation steps and better automation. Notably, methods for the direct reprocessing of plastics into valuable chemicals or polymers take advantage of metal catalysts (see Sections 3.2.3 and 3.3.3). Finally, coupling one-pot techniques with the use of lytic agents other than the conventional ones may remarkably enlarge the scenario of depolymerisation products beyond that of the simple plastic components. Table 7 summarises the most common monomers obtainable in the metal-catalysed depolymerisation of plastics described in the present review. It is predictable that a greater variety of added-value products, such as monomers, oligomers and the chemically derived, functionalised compounds thereof, may be obtained by developing alternative depolymerisation pathways and reprocessing strategies.

**Table 7:** Common monomeric products of metal-catalysed chemolytic depolymerisation reactions of plastics.^a^

polymer type	chemolytic method				

	hydrogenolysis	hydrolysis	alcoholysis	glycolysis	aminolysis

polyolefins	hydrocarbons^b^	—	—	—	—
polyesters	diols	acids (+ diols)	esters (+ diols)	esters	amides + diols
polycarbonates	diols + CH_3_OH	diols + CO_2_	diols + carbonates^c^	diols + cyclic carbonates^c^	diols + ureas^d^
polyamides	amines, alcohols	amines, acids	—	—	—

^a^The monomeric products indicated (e.g., diols) are those corresponding to the repeating units in the polymer. ^b^Usually liquid mixtures. ^c^Organic carbonates. ^d^N*-*substituted ureas.

In conclusion, as it was previously outlined, circular chemistry is a precondition for a truly circular economy, particularly in the field of production of goods and materials [351]. Chemical recycling via metal catalysts may effectively contribute to a circular recycling vision for postconsumer plastics, provided that the strategy is further developed and improved, aiming to reduce costs and environmental impact of selective depolymerisation processes. The design of novel versatile catalysts and more sustainable processes are key in this direction.
